# An Association of Cancer Physicians’ strategy for improving services and outcomes for cancer patients

**DOI:** 10.3332/ecancer.2016.608

**Published:** 2016-01-05

**Authors:** Richard Baird, Ian Banks, David Cameron, John Chester, Helena Earl, Mark Flannagan, Adam Januszewski, Richard Kennedy, Sarah Payne, Emlyn Samuel, Hannah Taylor, Roshan Agarwal, Samreen Ahmed, Caroline Archer, Ruth Board, Judith Carser, Ellen Copson, David Cunningham, Rob Coleman, Adam Dangoor, Graham Dark, Diana Eccles, Chris Gallagher, Adam Glaser, Richard Griffiths, Geoff Hall, Marcia Hall, Danielle Harari, Michael Hawkins, Mark Hill, Peter Johnson, Alison Jones, Tania Kalsi, Eleni Karapanagiotou, Zoe Kemp, Janine Mansi, Ernie Marshall, Alex Mitchell, Maung Moe, Caroline Michie, Richard Neal, Tom Newsom-Davis, Alison Norton, Richard Osborne, Gargi Patel, John Radford, Alistair Ring, Emily Shaw, Rod Skinner, Dan Stark, Sam Turnbull, Galina Velikova, Jeff White, Alison Young, Johnathan Joffe, Peter Selby

**Affiliations:** 1 ACP Executive Member; 2 ACP Strategy Drafting Group; 3 Supporting Chapter Author; 4 Senior Author; 5 Addenbrooke’s Hospital, Cambridge, UK; 6 University of Leeds, Leeds LS2 9JT, UK; 7 Edinburgh Cancer Research Centre, UK; 8 Wales Cancer Research Centre, Cardiff, UK; 9 Beating Bowel Cancer, Harlequin House, 7 High St, Teddington, Middlesex TW11 8EE, UK; 10 London Deanery, Stewart House, 32 Russell Square, London WC1B 5DN, UK; 11 Queen’s University, Belfast, UK; 12 Guy’s and St Thomas’s Hospital, London, UK and Medical Affairs Manager, Pfizer; 13 Cancer Research UK, Angel Building, 407 St John Street, London EC1V 4AD, UK; 14 Severn Deanery, Vantage Office Park Old Gloucester Road, Hambrook, Avon, Bristol BS16 1GW, UK; 15 Northampton General Hospital, Cliftonville, Northampton NN1 5BD, UK; 16 University Hospitals of Leicester, Infirmary Square, Leicester LE1 5WW, UK; 17 Queen Alexandra Hospital, Portsmouth, UK; 18 Lancashire Teaching Hospitals, UK; 19 Southern Health and Social Care Trust, Southern College of Nursing, Craigavon Area Hospital, 68 Lurgan Road, Portadown, BT63 5QQ, UK; 20 NIHR Biomedical Research Centre, Royal Marsden Hospital, London, UK; 21 University Hospitals Bristol, Bristol, UK; 22 Clatterbridge Cancer Centre, Clatterbridge Health Park, Clatterbridge Rd, Wirral, Merseyside CH63 4JY, UK; 23 Mount Vernon Cancer Centre, Northwood, UK; 24 Kent Oncology Centre, Maidstone, Kent, UK; 25 Royal Free and University College Hospital, London, UK; 26 Guy’s and St Thomas’ Hospitals, London, UK; 27 North Middlesex University Hospital, UK; 28 NHS Fife, Scotland, UK; 29 Brighton and Sussex University Hospitals, UK; 30 Leeds Cancer Centre, St James’s University Hospital, Leeds, UK; 31 Huddersfield Royal Infirmary, Acre St, Huddersfield, West Yorkshire HD3 3EA, UK; 32 Weston Park Hospital, Sheffield, UK; 33 St Bartholomew’s Hospital, London, UK; 34 University of Leicester, University Rd, Leicester LE1 7RH, UK; 35 Freeman Hospital, Newcastle, UK; 36 Chelsea and Westminster Hospital, London, UK; 37 Poole Hospital, Longfleet Rd, Poole, Dorset BH15 2JB, UK; 38 Royal Marsden Hospital, London, UK; 39 Guy’s and St Thomas’ NHS Foundation Trust, London, UK; 40 University of Bangor, Bangor, Gwynedd LL57 2DG , Wales, UK; 41 Beatson West of Scotland Cancer Centre, Glasgow, Scotland, UK; 42 University of Birmingham, Edgbaston, Birmingham, West Midlands B15 2TT, UK; 43 Royal Victoria Infirmary, Newcastle, UK; 44 University of Manchester, Oxford Rd, Manchester M13 9PL, UK; 45 University of Southampton, University Rd, Southampton SO17 1BJ, UK; 46 Southampton General Hospital, Tremona Rd, Southampton, Hampshire SO16 6YD, UK

**Keywords:** medical oncology, cancer physicians, cancer policy, cancer strategy

## Abstract

The Association of Cancer Physicians in the United Kingdom has developed a strategy to improve outcomes for cancer patients and identified the goals and commitments of the Association and its members.

## Summary

In the Association of Cancer Physicians’ (ACP’s) new strategy for medical oncology in the United Kingdom, we are taking a broad view of developments which will bring benefits to patients with cancer and identifying the contributions that we can make to achieving these goals. Our consultants and their teams have contributed substantially to improvements in cancer outcomes over the past 25 years. We are greatly encouraged that over 50% of UK cancer patients now survive their disease for 10 years or more. We are at a time not only of unprecedented acceleration of knowledge with regard to all aspects of cancer, but also of rapid change in terms of patient management and new therapies. To deliver better outcomes for patients, we must overcome challenges over the next decade, such as increased demand for cancer services and financial constraint in the NHS.

The first version of the ACP strategy was published on 13 July 2015. This second version is prepared as an update to reflect the publication of the Report of the Independent Cancer Taskforce entitled ‘Achieving World-Class Cancer Outcomes. A Strategy for England 2015–2020’ [1]. We have also responded in this strategy to helpful meetings with the President and the Senior Officers of the Royal College of Physicians. These inputs, arising soon after the initial publication of our strategy, were felt by the ACP Executive to be of sufficient importance to justify bringing forward the annual update of our strategic document.

The independent cancer taskforce (ICT) proposes the following six strategic priorities:

- Spearhead a radical upgrade in prevention and public health
- Drive a national ambition to achieve earlier diagnosis
- Establish patient experience as being on a par with clinical effectiveness and safety
- Transform our approach to support people living with and beyond cancer
- Make the necessary investments required to deliver a modern high-quality service
- Overhaul processes for commissioning, accountability, and provision

The ICT sets out its benefits potentially to be an additional 30,000 patients per year surviving cancer for 10 years or more by 2020 of which almost 11,000 will be through earlier diagnosis. In addition, it is hoped to narrow the gap between England and the best countries in Europe in cancer survival, increasing integration of health and social care, improve patient information and empowerment, improve patient experience and quality of life, reduce the growth in the number of patients being diagnosed with cancer and reduce the variability of access to optimal diagnosis and treatment and the consequent inequalities in outcome. This may be associated with significant savings through improving the process and outcomes of patient care which could be re-invested to achieve further improvements in future. We welcome the prospect of investment in aspects of cancer prevention, early diagnosis, management and the support of patients with cancer throughout their journey that has been identified by the ICT and will lend our support to those who are charged with arguing the case for this with Government.

We believe that the ACP’s strategy is compatible with these goals and that our commitments will contribute substantially to the delivery of the ICT goals.

We believe that our new strategy will further improve outcomes for our patients by guiding continued improvement in the quality of our own professional practice and help support collaborations with the many other disciplines and professions involved in cancer care. We are committed to working with our patients and colleagues to improve the outcomes for patients with cancer so that 75% survive for 10 years or more, with improved patient experience and quality of life, within the next couple of decades. To contribute to this vision, over the next 3–5 years, the Association of Cancer Physicians (ACP) will work to achieve the following three broad goals:

- **The delivery of excellent and safe medical oncology for all patients.** We will continue the development and strengthening of multidisciplinary, specialised and patient centred care. Improved patient care will be delivered by adequate numbers of highly trained medical oncologists, through engaging closely with patients to understand their needs, and informed by high-quality research and innovation. Increasing incidence of cancer means that patient demand for medical oncology services may outstrip our ability to provide enough clinicians to care for patients at the highest standards. This must be addressed by training, recruiting and retaining more medical oncologists and by developing innovative ways of working. Additionally, we will aim to support the ongoing education of our medical colleagues, allied health professionals and patients about cancer and its management. We will ensure that training standards remain high and that the discipline grows to a consultant number that can ensure safe and excellent cancer care for patients across the United Kingdom. We will grow the number of medical oncology consultants until there is more than one full-time equivalent (FTE) consultant for every 100,000 people in the United Kingdom, aiming to do so by 2020 and then approaching 1.5 FTE consultants per 100,000 people as soon as that is possible. This will require the right numbers of trainees so that the workforce is sustainable in the long term.
- **A substantial contribution to the overall development of NHS services** and help to cope with the challenge the NHS faces in dealing with acute cases and the medical problems of the aging population. We will do this by developing cancer services to cope effectively with these pressures and hence relieve pressure on other parts of the NHS. We particularly identify two areas of growth in the demand for cancer care, acute oncology and the care of older cancer patients. Most medical oncologists will increasingly engage in these areas. As a specialty, we will always take a flexible approach to developing our expertise and the contributions we make to healthcare in the NHS. Although we do not expect that consultant medical oncologists will undertake unselected general medical acute duties, acute oncology and improved care for older cancer patients will be a substantial contribution to the challenges faced by the NHS.
- **A substantial contribution to the development of innovative approaches to cancer care.** We will collaborate closely with primary care and all of the other relevant specialties and groups of health professionals, to develop new ways to provide access to high quality diagnosis, prevention and treatment. We will better exploit modern health informatics, and better support the rapidly growing number of cancer survivors. We will develop and support the rapid adoption of evidence-based innovations arising from biomedical sciences to create a more precise approach to oncology, providing patients with a higher probability of success and a lower probability of toxicity.

This strategy will be delivered over the next 3–5 years, but we will manage the strategy with annual review of progress - updating and resetting the objectives for each year. These goals will be delivered through 12 specific commitments. This work must be specific, measurable, achievable, realistic and timely and we are identifying a series of specific actions and measures for success, and leadership roles, for each of our commitments. Wherever possible we will use measures proposed by the ICT and link our performance to those requirements.

We will share and converge our goals and commitments with those of the 2015, ICT and with the other clinical and nonclinical bodies that are engaged in improving outcomes for cancer patients. We work as part of the Royal College of Physicians (RCP) [2] and work closely with all of the other relevant Royal Colleges. There is a special need to ensure that we work with colleagues in Clinical Oncology in the Royal College of Radiologists (RCR) and converge our strategies and share workforce plans [3]. We have shared our strategic planning with the RCR early and will agree joint working where appropriate. We will collaborate and consult with cancer charities, including those that focus on individual tumour types, and with Macmillan Cancer Support [4] and Cancer Research UK [5].

## Summary for patients

The Association of Cancer Physicians (ACP) is the professional organisation of medical oncologists. We are a major group of doctors providing care for patients with cancer. Our main area of expertise is in systemic anticancer treatments, including chemotherapy, biological and immune therapy, and new medicines that target-specific changes in a patients’ cancer (targeted treatments). In recent years, medical oncologists have contributed to the rapid improvements in cancer survival, with more than 50% of patients with cancer now surviving for 10 years or longer. Medical oncologists work in teams made up of people from different cancer care disciplines and professions. These teams ensure patients get the best treatment for their cancer to give them the best chances of survival and a good quality of life. We also have a strong tradition of research and innovation and are among the most research oriented of all medical disciplines.

In our new strategy, we set out our vision for the development of medical oncology. But we also indicate how we can make contributions to the overall improvement in cancer care for patients in the United Kingdom and contribute to the priorities set out by the Independent Cancer Taskforce (2015–2020) [1]. In particular, we show how we can help increase survival so that 75% of patients with cancer live 10 years or more within the next couple of decades, and improve patients’ quality of life and experience.

To ensure medical oncology continues to provide excellent care for increasing numbers of cancer patients, and improves cancer care, the ACP will take three broad approaches.

1. Improve the traditional strengths of medical oncology in providing excellent care by further engaging with patients to understand their needs, and having a strong emphasis on research and innovation. We will grow the number of medical oncology consultants until there is more than one full-time equivalent (FTE) consultant for every 100,000 people in the United Kingdom, aiming to do so by 2020 and then approaching 1.5 FTE consultants per 100,000 people as soon as that is possible. This will require the right numbers of trainees so that the workforce is sustainable in the long term.
2. Respond to the increasing pressures faced by the NHS caused by increasing number of people getting cancer, the complexity of treating older patients with patients and the pressures on all emergency services. We will work to strengthen cancer services – particularly acute oncology services that are there to immediately respond to the needs of patients – to ensure all patients get the treatment they need quickly. The ageing population means that there will be more patients with cancer and older patients tend to have other conditions as well as cancer. We must ensure the care older patients get is of the highest quality, appropriate for their level of fitness and their choices. Treatment should not be determined simply on the basis of age. To achieve this, all medical oncologists will contribute to service development, and we will support more oncologists in the future to develop special interests in the problems faced by older patients with cancer.
3. Develop and promote new ways of working and exploit new evidence-based innovations arising from research to improve cancer services. For example, we will:
  - work to better integrate primary care (for example GP services) and hospital cancer services to ensure quicker access for patients;
  - improve the use of computer technologies for better communication between doctors and with their patients and better record keeping;
  - analyse large sets of data, under careful conditions of confidentiality and regulation, to identify where improvements in care can be made;
  - better support the increasing numbers of cancer survivors who will require long-term support;
  - use findings from medical research to develop a more precise kind of oncology, providing treatments that are more likely to benefit patients and less likely to produce side effects.

We plan a series of actions to address these needs. We will also work closely with other organisations including Royal Colleges, charities and the NHS, to bring about the benefits envisaged in its new strategy.

## Figures and Tables

**Table:** 

CONTENTS	PAGE(S)
Summary	3–4
Summary for patients	4–5
Context	6
Introduction	7–8
Our goals, commitments and actions	8–14
How will this strategy be implemented?	14–15
An Association of Cancer Physicians’ strategy for improving services and outcomes for cancer patients—supporting chapters	16–58
