# Supplementary material for: An Association of Cancer Physicians’ strategy for improving services and outcomes for cancer patients
Source: Ecancermedicalscience. 2016 Jan 5;10:608. doi: 10.3332/ecancer.2016.608 (PMC4762575; doi:10.3332/ecancer.2016.608)
Supplement: Related texts

# Brief report

**Published:** 2016

## Context

This ACP strategy reflects a significant evolution of medical oncology planning and practice in the United Kingdom. Previous strategies necessarily focused upon the development of medical oncology as a research-based discipline whose main contribution to cancer care was the development and delivery of systemic anti-cancer therapies. In the last three decades, the specialty has undergone rapid expansion, both in consultant numbers and in the roles performed by medical oncologists. This requires a reassessment of our priorities and our role in providing the best cancer care and outcomes for our patients. Despite recent growth, the number of medical oncology consultants per head of the population in the United Kingdom is relatively low by European standards, and there is an increasing recognition within the United Kingdom that, despite substantial progress, the job of improving cancer outcomes is far from complete. Hence, our discipline is likely to continue to be one of the most rapidly growing of all medical subspecialties.

At this stage in the development of our discipline, we felt that we should shift the emphasis of our strategic planning, in order to:

- Continue to focus on our primary duty of improving survival and quality-of-life outcomes for our patients, by exploiting the remarkable opportunities provided by cancer research and innovative and excellent cancer care.
- Work with cancer patients in the production of the strategy.
- Provide leadership in specific areas of existing and emerging strength of the ACP and its members. In the United Kingdom, as in the rest of Europe, healthcare is under tremendous pressure. Increasing service demands, an ageing population and increasing costs are all generating pressure on NHS services. Medical oncology is now big enough and strong enough as a discipline to make a substantial contribution, providing leadership and support for our many colleagues in all of the healthcare professions in the management of the pressures in cancer care. Examples include the following:
  - Delivery of systemic therapy – we will be major providers of healthcare and lead innovations and service developments.
  - Acute oncology services, which often include responsibility for the selected intake of patients into the hospital where they have cancer and cancer-related problems. This will relieve pressure on the unselected intake of ill patients that places huge demands upon NHS services in the United Kingdom.
  - Oncology care for older patients with cancer. The ageing population generates ever-increasing pressures on the healthcare system and we believe that medical oncologists can make a major contribution in this area which should help the NHS as a whole.
- Act as strong advocates of improvements and contribute in significant ways in other areas which improve outcomes and the quality of the patient experience. For example, although medical oncologists are rarely the doctors who make the first diagnosis of cancer, we will strongly support and value the moves towards earlier diagnosis of cancer.
- Retain a strong focus on research and innovation. We summarise these contributions in the strategy and we believe that they will be crucial in improving outcomes in the coming years.

The emphasis on outcomes and on the broad developments in cancer diagnosis and care are a significant evolution from previous practice. They have been warmly welcomed and endorsed by all of our members and the leaders of our specialty in the United Kingdom. The strategy is evidence based with 12 Supporting Chapters covering our commitments. We have invited input from experts in each area including patients and those from other disciplines.

Many of the broad range of recommendations of the Independent Cancer Taskforce in the United Kingdom for improving cancer diagnosis and cancer care in the United Kingdom are endorsed and integrated into our strategy. By working very closely with the many other healthcare professionals, researchers, health service managers and, in particular, with patients, in implementing the recommendations of the taskforce, we look forward to making a crucial and increasing contribution to improving cancer outcomes substantially in the coming decades.

This strategy is a living document. Its changes in emphasis have been debated carefully for more than a year. We will review it annually and assess our progress against the commitments we have made and the measures we have indicated. We hope to be able to show steady progress in the outcomes for our patients and demonstrate the value of the ACP’s increasing contributions, as the specialty of Medical Oncology continues to grow in coming years.

## Introduction

We have seen great improvement in the survival of patients with cancer in the United Kingdom in recent decades. More than 50% of all patients with cancer are alive 10 years after their diagnosis, a doubling in survival since the 1970s (Figure 1). Nevertheless, the care of patients with cancer remains a huge challenge to healthcare systems in the United Kingdom and globally, which face twin issues of increasing cancer incidence and improving survival. The ageing population in the developed world and the increasing exposure of populations in the developing world to environmental carcinogens mean that cancer incidence will rise. The exciting opportunities presented by advances in biomedical science and applied health research are a huge opportunity to improve further cancer treatment, earlier diagnosis, screening and prevention. However, such improvements present a massive challenge in terms of the quality, quantity, and cost of care that can be provided.

Medical oncology arose as a discipline in response to changing needs of patients with cancer for systemic anticancer therapy. As a discipline, it has a long and strong tradition of responding to patients’ needs by changing its shape and patterns of practice, training programmes, and continuing professional development. Medical oncologists are committed to improving outcomes for patients with cancer and survivors. As a specialty, we principally deal with patients who already have a cancer diagnosis, although we have substantial input into public awareness, investigation and early diagnosis, and issues around access to care, throughout the patient’s journey.

Among the many important outcomes, cancer survival is probably the one that most clearly reflects the activities of medical oncologists. We are committed to continuing improvements in survival in coming decades that will improve 10-year cancer survival for the overall cancer population from its current level at 51% towards 75% in 2035. We will work towards this ambition but as a discipline we are mindful that survival to and beyond 10 years does not tell the whole story for our patients. We are committed to ensuring the patient has the best experience of diagnosis and treatment that is possible with a strong focus on maintaining quality of life throughout their journey. A patient-centred approach includes excellence in communication and close integration with many other disciplines that also play important roles throughout their care.

Multidisciplinary and multiprofessional teamworking with clinical and surgical oncologists, oncology nurses, clinical researchers, pathologists, radiologists, managers, and many others has been a key part of the progress made for cancer patients and must be sustained and improved. Specialisation of all oncologists into a small number of cancer sites is part of the success of multidisciplinary teams (MDTs).

Appropriate liaison with palliative care teams represents a continuing fundamental role of the Medical Oncologist. We welcome the emphasis given by the independent cancer taskforce (ICT) [1] on the improvement of quality of life in patients after treatment and at the end of life. The orchestration and implementation of carefully planned, integrated, holistic care, coordinated at an appropriate point following diagnosis, is recognised to be key. Additionally, our commitment to continued improvements in joint working with palliative care colleagues in end of life planning and management, will support better patient care and experience and help prevent avoidable hospital admissions.

The NHS strategy for cancer has been the subject of a series of valuable reports [2]. In 2015, the report of the ICT informed and will influence the work of medical oncologists. The key priorities of the taskforce are listed in the summary above and are all compatible with the plans and goals of medical oncologists and the Association of Cancer Physicians.

Medical oncologists are actively involved in developing new, updated approaches to systemic anticancer therapy (SACT) [3, 4]. From April 2013, chemotherapy services have been directly commissioned by NHS England. The chemotherapy service specifications for NHS England were implemented on 1 October 2013. A key requirement of these specifications is that all providers of chemotherapy services have in place an electronic prescribing system. Our strategy is UK wide in its scope and we anticipate considerable changes in the NHS in the UK provisions of systemic therapy for patients with cancer in coming years and we will work to advise on, develop, optimise, and deliver these.

We will work closely with the Royal Colleges to contribute to the development of the ‘Future Hospital’ model. We agree with the Royal College of Physicians (RCP) that:

‘Patients should have access to the care they need, when they need it. Many patients can be managed well in primary care, but most will need specialist help at some point. Some people’s needs may be met by delivering specialist care in new ways into the community. However, being admitted to hospital will be essential for others. Barriers to accessing early expert care must be removed. Specialist medical care should reach from wards into the community. Swift access to expert diagnosis and treatment improves outcomes for patients and can result in long-term savings. Supporting patients to recover and manage their conditions must be a priority in all policies’.

The new ACP strategy draws on our involvement and support for these ongoing national initiatives. We have characterised the objectives that are most relevant to the ACP and developed a series of commitments which relate to the core activities of medical oncologists and our contribution to increasingly important developments in cancer care.

The ACP is updating its constitution and ways of working to strengthen the capacity of the Executive, add stronger patient and public representation, and form stronger regional links. We will establish leadership roles and working groups to take forward all of our commitments. This will strengthen the implementation of our strategy in the coming years.

## Our goals, commitments and actions

### Goal 1

***The delivery of excellent and safe medical oncology for all patients through the continued development and strengthening of multidisciplinary specialised and patient centred care, informed by high-quality research and innovation, engaging closely with patients and delivered by adequate numbers of highly trained medical oncologists.***

This goal will be delivered through the following six commitments.

**Commitment 1.** Deliver excellent, evidence-based, multidisciplinary care to ensure the best possible quality of life and survival and promote equal access across United Kingdom.

In the United Kingdom, medical oncologists and our colleagues have made substantial progress in the development and delivery of expert multiprofessional specialised care for patients with cancer. This has contributed to improved outcomes. Once diagnosed, patients with cancer are usually now receiving care in the United Kingdom of comparable quality to that received in the best among other European countries. Sustaining and strengthening multiprofessional specialised care is vital if we are to achieve our shared commitment to increase survival for patients with cancer to 75% by 2035, uphold patient centred and high-quality care, and continue to improve the patient experience. The increasing complexity of cancer care as it develops greater precision, which means that treatments are deployed in a stratified and personalised way and will increase demands for specialisation. Continuing professional development to sustain that specialisation throughout the career of a medical oncologist is needed. Sustaining existing multiprofessional working and appropriate levels of specialisation in a small number of tumour sites is key to continued success. However, the number of cancer sites on which oncologist specialise may have to be reduced. We believe that in future, sustained cancer site specialisation will need to be strengthened by senior clinical advisor/mentor system for consultants taking up their first post at this level. This will provide a supportive framework for consultants who have a comprehensive training in oncology but limited experience in a cancer site in which they will specialise in their consultant post. This policy is endorsed by the Royal College of Physicians and is in keeping with wider implementation of consultant mentoring for new consultants.

We welcome the endorsement of multiprofessional working and multidisciplinary teams given by the ICT and especially welcome the emphasis given to the role of Clinical Nurse Specialist in ensuring patients receive excellent care and have a positive experience of that care. There are unacceptable disparities in access to care across United Kingdom. For example, Northern Ireland, Wales and Scotland al lack a cancer drugs fund (CDF), present in England, which reduces access of patients in these areas to some new drugs and also clinical trials. The ACP will use its expertise and influence in arguing strongly that such disparities must be addressed.

#### Actions

- A new early consultant senior clinical advisor/mentor system for site specialised cancer care
- ACP workshop on Precision Oncology completed in October 2015, and we will publish an ACP Problem Solving textbook in 2016
- Use our expertise and influence to address disparities in care across United Kingdom

**Commitment 2.** Sustain a strong, patient-centred focus that influences all aspects of practice throughout patients’ lifetime with and after cancer, often using patient reported outcomes.

Medical oncologists have prioritised the development of patient-centred approaches and many have been at the forefront of developing the concepts and knowledge base which has underpinned successful patient centred care. The improvement of communication skills, the use of routine collection of quality of life data and Patient Reported Outcome Measures, and active patient and public involvement in cancer care, are all examples of areas that need to be sustained and improved. We believe that the development of innovative treatments and of accessible informatics approaches will allow us to strengthen our commitments to patient centred care.

#### Actions

- Continue to incorporate substantial training and CPD in advanced communication skills
- Promote the strengthening of psychosocial support for cancer patients in cancer services across United Kingdom
- ACP workshop on Patient Centred and Integrated Cancer Care in October 2016 with an ACP Problem Solving textbook in 2017
- Review/workshop of existing electronic systems for patient toxicity reporting to develop recommendations for best practice and wider implementation

**Commitment 3.** Continue our commitment to conduct high-quality research and innovation to answer key questions that will determine optimal patient management. Our work will help to ensure the translation of new knowledge from the laboratory to the clinic and from clinical evidence into clinical practice.

We will commit to sustaining the role of UK medical oncology as a research-intensive discipline, at the forefront of biomedical and health research in the United Kingdom and internationally. There are exciting opportunities for research and innovation to improve health service delivery and treatment for patients with cancer in fields, including immunotherapy, personalised/precision medicine based on rapid advances in genomics and proteomics, chemoprevention, and the novel methodologies based on rapid advances in informatics and biostatistics.

We welcome the ICTs identification of the importance of access to innovative drugs, including immunotherapies, and state-of-the-art/innovative approaches to the delivery of systemic therapy close to patients, through electronic prescribing and with excellent underpinning informatics. Stratified approaches to drug treatment will be central to the future of oncology and to the work of members of the ACP.

#### Actions

- A new ACP Executive Academic lead
- Increased contributions to national cancer research meetings
- All ACP members will contribute to clinical trials which are appropriate to their institutions and the level of team support

**Commitment 4.** Produce an expert workforce through a well-planned, nationally coordinated training programme to address knowledge, skills, and competency, with excellent Continuing Professional Development and a robust and supportive trainee and young consultant advisory/mentor system.

Medical oncologists and the Association of Cancer Physicians take considerable pride in the quality of the training provided to young doctors who are the medical oncologists of the future. This training is under considerable pressure, because of the increasing complexity and scale of oncological care, the increasing age of cancer patients with attendant comorbidities, and the complexities of regulation and oversight of practice and research. Demands are also being placed upon specialists to support currently inadequate acute general medical care provided in the NHS. (REF: Hospitals on the Edge? The time for Action. RCP 2012, Shape of Training Review. http://www.shapeoftraining.co.uk/static/documents/content/Shape_of_training_FINAL_Report.pdf_53977887.pdf)

Our training programme must sustain its focus on producing cancer specialists of the highest quality with skills that include the ability to develop their services and conduct research and innovation. We see a growing requirement for strengthening and refining the training and continued professional development of specialists in each of their chosen areas of cancer care and their specific disease sites. We will strengthen this through our senior clinical advisory/mentor system. The delivery of acute oncology – our major, rapidly growing commitment to the provision of acute care for patients with cancer – means that it is also important to sustain our skills in general oncology and general internal medicine. We must ensure the acute oncologists meeting patients are fully conversant with the needs of their patients who have complications of treatment or complications of cancer, which often result in common medical conditions, such as infection, breathlessness, pain, or metabolic imbalance.

Consultant medical oncologists will take a flexible approach to their work and to the needs of the wider health service. They will contribute to the future Hospitals acute workload through their contribution to Acute Oncology and the associated selective intake of patients with cancer related problems and their appropriate contribution to the care of all cancer patients. However, in almost all job plans, this will not be through acute unselected intakes of patients that will remain exceptional for medical oncologists. To support our expanding role in acute and elective care of patients with cancer, we will enhance our training and CPD in the care of older patients with cancer, cancer survivors and in precision oncology.

The ICT has recommended joint planning of consultant and trainee numbers between medical oncologists and clinical oncologists. We strongly support this ambition and we will, therefore, reinforce our prior commitment to a joint planning process and working party with colleagues from the College of Radiologists in clinical oncology.

#### Actions

- A working party (2015–2016) on workforce planning and training numbers jointly with clinical oncologists
- Acute oncology service developments, training and CPD (published text 2014)
- Training and CPD to address the problems of older patients with cancer (published text 2016), precision oncology and cancer genetics (Workshop October 2015)

**Commitment 5.** Ensure that the growth of medical oncology matches the needs of our patients and future cancer services, with the flexibility to adapt to changing needs/demographics.

Demographic change, the aging population and the increasing number of patients with cancer require a proportionate increase in the number of consultant medical oncologists matched by appropriate training numbers. This absolute requirement is an essential part of our strategy and without it the quality of care for our patients will inevitably suffer. There are many pressures that are increasing demands for medical oncologists including: the complexity of care; the complexity of healthcare regulation; economic austerity and its impact on healthcare; and the deployment of sophisticated tests alongside individualised treatment plans. Specific pressures include the development of acute oncology with its associated staffing requirements. We lag behind several European countries in our provision of medical oncology and the United Kingdom has fewer medical oncologists per patient with cancer than any other European country providing data to the European Society for Medical Oncology [5], and there are also challenges in the provision of radiotherapy staffing [6].

We believe that medical oncology consultant numbers need to grow to 1.0 FTE per 100,000 UK residents by 2020 and to 1.5 FTE per 100,000 UK residents as soon as possible after 2020, and be evenly distributed across United Kingdom, which means the number of consultants will grow to a total of over 900 full time equivalents. Where medical oncologists take on roles in oncology service developments in addition to delivering SACT, then specific additional funded protected time and support teams will be essential.

#### Actions

- Establish a joint working party on workforce planning with the Royal College of Radiologists (Commitment 4). We will establish, with colleagues from clinical oncology, the National requirement for WTE consultants engaged in delivery of systemic therapies for cancer, now and for the future. We will be cognisant of the need to expand the workforce in radiation oncology also.
- We currently believe that this will require at least 1.0 FTE consultant medical oncologists per 100,000 UK residents by 2020 to ensure safe and effective delivery of systemic therapy and to improve and deliver many other aspects of excellent cancer care. This is likely to need to increase to 1.5 FTE consultants per 100,000 people as soon as that is possible.
- Ensure that medical oncologists who contribute to broader aspects of care (e.g. acute oncology, survivorship) have sufficient protected time for this work, in addition to work to deliver systemic anti-cancer treatment.

**Commitment 6.** Increase patient engagement in the work of the ACP and the delivery of cancer care.

Patient and public involvement in the development of cancer services and in the delivery of care to individuals has grown and become more effective in recent years. The ACP welcomes and supports these developments. We will formalise and increase patient involvement in our work. The ACP has involved patients in the drafting of this strategy and will do so in its future work. Two patients were appointed to contribute to the drafting of this strategy and will become full members of the ACP Executive.

#### Actions

- Develop a new ACP Patient and Public Engagement (PPE) group and establish support and training for PPE in the work of the ACP

### Goal 2

***A substantial contribution to the overall development of NHS services and help to cope with the challenge the NHS faces dealing with acute cases and the medical problems of the aging population.***

This goal will be delivered through two commitments.

**Commitment 7.** Complete the development of a comprehensive Acute Oncology service nationwide, including provision of timely and dedicated care for patients whose primary cancer site is unknown.

Patients with cancer being cared for in the community or by general healthcare services frequently need prompt access to specialised oncology to ensure swift diagnosis of their disease, and of complications arising from the disease or its treatment. There is evidence that such access has been slow in the United Kingdom in the past and innovative service development is ongoing, with medical oncologists playing a leading role, to meet that essential challenge. Acute oncology services are multi-professional in their nature and collaborations with many oncology disciplines and with general services are essential. However, much of the consultant and trainee commitment to acute oncology will be provided by medical oncologists. The development of these services to a high standard and their implementation across all of the United Kingdom is an important strategic commitment for the Association of Cancer Physicians to make its contribution to acute medical care. The ICT has identified the importance of acute oncology and the importance of evaluating its systematic development across the country.

#### Actions

- Complete an acute oncology service specification in 2015 and work to deliver a nationwide acute oncology service
- Continuing the development of training and CPD in acute oncology (published text 2014)
- Develop the national network of expertise in acute oncology

**Commitment 8.** Almost all medical oncologists will continue to be involved in the care of older patients with cancer. We will work to develop improved care for older patients with cancer closely with geriatricians and all other healthcare disciplines and professions.

The ICT has noted the importance of providing appropriate high-quality cancer care for older patients with cancer. The ACP has already identified this as a high priority and we now have completed the preparation of the ACP text on problem solving in older patients with cancer jointly with the British Geriatrics Society, which will be published within a few months. Some medical oncologists will take specific leading and organisational roles to address the important issues within cancer care for older people, such as the evaluation of frailty and concomitant morbidity. These roles should have support and protected time to ensure service developments and drive continuing professional development. As researchers, we would wish to provide leadership in the evaluation and development of therapies in older patients, supported by the National SACT database, currently unique in national cancer management.

#### Actions

- Publication of the ACP ‘Problem Solving in Older Cancer Patients’ book (2016)
- Lobby for a national initiative to develop innovative services for older cancer patients and develop new roles for oncologists who will take a specific interest and support and advise their colleagues in these areas of work
- Support developments for older patients with cancer in Research and Innovation, Education and Training and strengthen national and local collaborations

### Goal 3

***A substantial contribution to the development of innovative approaches to cancer care.***

This goal will be delivered through four commitments.

**Commitment 9.** Support new ways of working and the reconfiguration of services in order to provide the best and most timely access to investigations, prompt diagnosis and treatment by the appropriate specialised team for all patients.

The timeliness of diagnosis and access to high-quality cancer care remains a challenge to UK health services and innovative approaches are needed. There is evidence that some of the shortfall in survival in the UK results from delays in access to diagnostic services leading to specialised cancer care for patients. This results in the presentation of patients with more advanced disease which in turn contributes to reduced one-year survival. Medical oncologists are usually not ‘doctors of first contact’ for patients with symptoms that may be due to a cancer, but they do have substantial influence over the configuration of services and access to their services. We are committed to work with colleagues in primary care and in other hospital based services to speed the timeliness of diagnosis and improve access.

There are many fresh approaches to be considered. Closer integrated working with primary care in cancer centres and cancer units has always been a stated goal of the Association of Cancer Physicians and of the strategic documents generated by Government and voluntary sectors. However, progress is incomplete and has been slow. New technologies, especially in health informatics, should improve opportunities to integrate care. Medical oncologists will be at the forefront of advocating and implementing these in their cancer care communities, to produce an integrated approach to prompt access and delivery of excellent care.

New ways of working, envisaged in the ACP strategy, will converge with the new approaches to diagnostic pathways laid out by the ICT. The ACP has not been a prime mover in new diagnostic pathways but closer involvement of oncologists with primary care colleagues in the implementation and promotion of these plans is likely to strengthen the case and improve the probability of their adoption across the NHS. We will look for ways to directly support primary care in knowledge development and in the development of pathways for assessment and referral of patients with suspected cancers.

#### Actions

- ACP workshop on patient centred and integrated cancer care (October 2016)
- Support local initiatives for integrated service developments

**Commitment 10.** Work to improve care through the development of appropriate state-of-the-art health informatics.

Medical oncologists recognise the importance of appropriate information in delivering both high quality clinical care and high-impact clinical/academic research in patients with cancer. The ACP believe that through the increased use of information tools, we can enable change which leads to better patient safety and improve patient experience. Systems can improve patient engagement in their care and provide more cost-effective resource utilisation. They can improve patient pathways and provide more responsive audit and performance management, newer avenues for clinical research and reduced administration time for clinical staff.

Ultimately, the ACP wishes to see the development of development of electronic health records for all cancer patients. With appropriate security, data governance and consent, we will work to ensure the effective sharing of this data with other members of the integrated care team across different healthcare organisations and between different software platforms. We wish to see the patient become an active user and ultimately contributor to the electronic health record through patient portals and the use of web-based software accessible through any enabled device. We will encourage clinic teams to exploit the opportunities offered by patients to communicate with the clinical team using video-conferencing, the completion of relevant electronic questionnaires and the use of devices to monitor patients in their home. We believe this will allow the development of new models of care for cancer patients on treatment, during follow-up and for the long-term monitoring of outcomes and toxicity.

The data model that underpins these activities must be agreed nationally to ensure that data can be collected once and used across multiple applications. New technology including natural language processing and self-learning analytic software will also be exploited to maximise the potential of the data collected through the delivery of routine health care.

#### Actions

- ACP workshop on Health Informatics in Cancer Care 2016
- Strengthen research, governance and leadership in Informatics in medical oncology

**Commitment 11.** All medical oncologists will make contributions to the planning and delivery of services for cancer survivors and some will develop special interests in this area.

We will promote the development of roles for individuals who take special responsibilities in this area to promote service developments, work with the many other disciplines and professions who will also contribute, and work together nationally to plan appropriate developments in this area. Such posts will require appropriate support and protected time. Oncology has led the way in transitional care and is already providing a number of late effects clinics in younger patients that can be a model for practice across age groups.

#### Actions

- Develop new oncology roles with special interest to develop services for cancer survivors
- Contribute to the Medical Oncology-led RCP conference on survivorship and late effects in 2016

**Commitment 12.** We will develop precision oncology that will allow the development of cancer treatments with a greater probability of success and a lower probability of toxicity.

Rapidly growing knowledge of the genetics and biology of cancer and environmental factors, which influence the incidence and outcomes of cancer, must be incorporated into research and into new strategies to prevent and manage cancer more precisely. This includes families at high risk of cancer.

Rapid strides in sequencing technologies now are providing insights into the somatic genetics of many cancers. We are moving towards the using these technologies to individualise and stratify cancer diagnostics and therapeutics resulting in a much more precise oncology practice. The use of somatic genetic studies to determine the choice of the therapy for cancers is now a well-established clinical practice in haematology and solid tumour oncology including paediatric oncology. Many of the exciting new targeted therapies for cancer are applicable only to patient populations who have a defined somatic genetic abnormality in their tumours.

The genetic basis of most familial cancer syndromes is now substantially understood and studies of their germline genetics, of individuals and families, are a vital part of the management of these relatively uncommon but vitally important disorders. Genetic epidemiology is now informing our understanding of factors in the causation of many cancers. This explosion of knowledge and its immediate clinical application must be built into service provision and into the training and continuing professional development of all oncologists. We propose to work with colleagues in clinical genetics and other related disciplines to strengthen these themes within medical oncology in the United Kingdom.

#### Actions

- ACP workshop on precision oncology and genetic factors completed in October 2015 with an ACP textbook on the subject agreed and funded in 2016/17
- Development of the medical oncology training programme in genetics
- Evaluation and development of online support systems for precision practice for all oncologists

## Figures and Tables

**Figure 1.** There has been a steady increase in the survival of cancer patients at one year, five years and at ten years between 1971 and 2010/11. Impressive and continuing increases in survival at one year and at five years are apparent. In particular, the survival of cancer patients in the UK ten years after their diagnosis, which will equate to cure for most of them, has increased from 25% in 1971 to over 50% in 2010/11.

## References

[1] “Achieving world-class cancer outcomes (2015) A strategy for England 2015–2020 Independent cancer taskforce”. www.cancerresearchuk.org/sites/default/files/achieving_world-class_cancer_outcomes_-_a_strategy_for_england_2015-2020.pdf.

[2] A policy framework for commissioning cancer services A report by the expert advisory group on cancer to the chief medical officers of England and Wales, 1995 (The Calman-Hine report).

[3] National chemotherapy advisory group Chemotherapy ervices in England: ensuring quality and safety Department ofHealth.

[4] National audit office (2010). Delivering the cancer reform strategy, Department of Health.

[5] de Azambuja E, Ameye L, Paesmans M (2014). The landscape of medical oncology in Europe by 2020. Ann Oncol.

[6] Lievens Y, Defourny N, Coffey M (2014). Radiotherapy staffing in the European countries: final results from the ESTRO-HERO survey. Radiother Oncol.

# Brief report

**Published:** 2016

## How will this strategy be implemented?

The Association of Cancer Physicians is a professional organisation and can only bring about the developments and changes envisaged in this strategy through the actions of its members and the influence it can exert over other bodies, especially the National Health Service. We envisage a series of implementation approaches:

- ACP members can use the strategy, and its continuing year-by-year development, as a resource at all stages of their careers, in the development of their practices, in the influence they bring to bear upon their local organisations and in their national and international roles.
- Patients with cancer are key to the implementation of this strategy. The patient voice will be heeded not only by healthcare professionals and NHS managers but also by the wider community and political leaders. The partnership between patients and the Association of Cancer Physicians will greatly strengthen our ability to see this strategy implemented.
- The Association has formal responsibilities for the development and oversight of training, continuing professional development and the support of the careers of our members. Many of the specific actions which are envisaged as a consequence of this strategy can be delivered by the Association in this role. Recent examples include the development of workshops and problem-solving books to strengthen the professional base and awareness of issues in acute oncology, and cancer in older people. This implementation mechanism has already been agreed for 2015/16 to be applied to precision oncology, drawing on advances in biomedical sciences to improve the effectiveness and reduce the toxicity of treatments. There will be a rolling programme of these exercises and workshops focused on aspects of implementing the strategy.
- The Association will form strategic alliances for specific pieces of work. Our colleagues in clinical oncology in the Royal College of Radiologists are our natural collaborators and allies in implementing improvements in the non-surgical aspects of multidisciplinary management of patients with cancer. We work closely and contribute substantially to the work of the major cancer charities and are grateful for their support already in the development of implementation strategies, workshops, and books through the ACP. Our members have local, regional, and national influence over the NHS from the commissioning and the provision of cancer services.
- The ACP will increase its international alliances and draw on the strengths of medical oncology in Europe and North America where, for example, advances in the care of older patients have achieved greater clarity and standing among oncologists. Knowledge management in oncology is not a national activity; it must be international, drawing on the results of clinical research and biomedical and healthcare research across the globe. We have recently formally formed a link with the European Society of Medical Oncology with a specific shared membership scheme.

We will review the Strategy, commitments and actions annually and devise a delivery plan for each year. In the first year (2015/2016), we will

- disseminate the strategy to oncologists, cancer care professionals, patients, and cancer organisations
- convene a working group and report on a workforce development plan (jointly with the RCR) to identify essential growth in consultant and trainee numbers
- establish a PPE group and deliver and update the ACP PPE plan
- convene a new executive committee with PPE representation and new leadership roles
- produce a plan for a senior clinical advisory/mentorship plan for new consultants
- publication of problem solving for older patients with cancer, a review of electronic toxicity reporting, the acute oncology service specification from the chemotherapy CRG and conduct workshops on precision oncology, integrated care, and in health informatics

# Brief report: An Association of Cancer Physicians’ strategy for improving services and outcomes for cancer patients—supporting chapters

**Published:** 2016

The 12 Supporting Chapters relate to each of our commitments and have been prepared by ACP members who specialise in these topics jointly with experts from outside the ACP who have generously given us their expertise and time. They are discussed as follows:

## 1. Multidisciplinary specialised care

**Professor Johnathan Joffe, Dr Adam Januszewski, and Professor Peter Selby**

Deliver excellent, evidence-based, multidisciplinary care to ensure the best possible quality of life and survival, with equal access across the United Kingdom.

Multidisciplinary teams (MDT) bring the expertise of all of the oncology disciplines to bear on the planning for best patient care and excellent MDT working is central to the whole-patient pathway. In the absence of multidisciplinary working, and consideration by a multidisciplinary team, a patient may be at risk of an inappropriate treatment selection or recommendation. For this reason, sustaining specialised, scientifically robust and excellent oncological care, has to remain central to the future of oncology. Oncology is a multiprofessional discipline bringing together nurses, doctors from diagnostic and therapeutic specialties, all of the allied health professions, health service managers and general medical and surgical services and primary care services. The skills mix for each component of this multiprofessional spectrum of care is evolving.

Outcomes for patients with cancer in the United Kingdom over the last decades have lagged somewhat behind relevant comparators across the world. In the 1990s, analyses suggested that one of the shortfalls lay in the provision of multidisciplinary specialised care [1–5]. Through a combination of government-driven and professionally-led initiatives, cancer physicians have played a major role in creating specialised multidisciplinary care which is one of the factors associated with the improving outcomes. All patients with cancer now receive expert, peer-reviewed, multidisciplinary specialised care delivered through multidisciplinary team (MDT) meetings and overseen by rigorous peer review provided through a process developed by cancer networks [6].

The simultaneous introductions of multidisciplinary teams, of greater specialisation in individual cancer sites, and the reconfiguration of services make it impossible to separately measure the impact of each component of the changes. However, the changes altogether have been a major factor in the increase in median survival for UK cancer patients from three years in 1995 to five years for patients diagnosed in 2008. The proportion of all cancer patients who survive for 10 years has increased to over 50%. Cancer Research United Kingdom has analysed one-, five-, and 10-year survival for England prior to 1995 and in five-year cohorts up to 2010 for the 21 most common cancers [7]. Substantial increases in survival of over 5% at 5 years are seen in 16 cancer sites, including the common cancers of breast (13% increase), colorectal (12% increase), and prostate (over 15% increase). Important exceptions are cancers of the pancreas (2% increase), lung (4% increase), and brain (4% increase) where even specialised treatments have not led to substantial improvements; testicular cancer where 5-year survival is 97%; and bladder cancer that has not changed. Although other factors (including novel therapies) have contributed substantially, epidemiological studies and expert opinion have attributed a significant proportion of that improvement to multidisciplinary specialised care, peer-review-driven service delivery and improved healthcare practices [8, 9].

The characteristics of an effective MDT in the United Kingdom setting have been well described [10] and the practical barriers to their success have been identified and valuable policy statements now exist [11]. Further developments to improve links across all professions and to primary care are being explored.

There has been extensive international experience and consideration given to multidisciplinary team working [12–18], but there is considerable international variation in practice [19]. The literature contains some evidence of improvements in outcome as a result of multidisciplinary cancer care [14, 19] and the need for further development [11, 15–18]. A systematic review suggested that there is a moderately consistent association between multidisciplinary care and patient survival but could not assert a causal association because of methodological limitations in the literature [20].

It is important for multidisciplinary teams to have a clear conceptual framework for their operation. Modern oncology practice requires that the patient is involved in major decisions. Therefore, the MDT, usually conducted in the absence of a patient, cannot take final decisions. MDTs present their members with the opportunity to achieve a consensus about the best option or possibly a number of acceptable equivalent options for the patient. They must recognise that some patient’s personal circumstances and choices may lead to the preference for a treatment that may not offer the very best potential for long-term survival. Multidisciplinary teams, therefore, present a consensus view of the recommended treatment and treatment choices that must be conveyed back to the patient by their individual oncologist at the time of their consultation. At this point, patient choices and preferences are included. Models in which MDT decisions are seen to be fixed recommendations that are handed to patients without consultation or consideration of their choices and preferences are undesirable [21–23].

We believe that retaining specialised multidisciplinary care with cancer physicians focusing on a small number of tumour sites in their overall job portfolio is critically important to sustaining improvement for cancer patients.

In order to retain and continue to improve the outcomes for patients with cancer, we must preserve the contribution of cancer physicians to multidisciplinary specialised care and preserve the multidisciplinary team’s contributions of key inputs to ensure the best outcomes for patients. There is currently a maximum specialisation for medical oncologists of three cancer sites and we expect this to be reduced to 2 or less in the next 5 years.

MDTs must continue to develop. Evidence suggests that to be reviewed in an MDT at a site where all specialities are represented means that patients are more likely to receive radical intervention. It is therefore important that the multidisciplinary team are present not only in the initial decision-making process of the MDT. Centralisation of service will improve access to the full MDT. However, to improve outcomes in oncology, we have to increase the number of patients treated appropriately. One way to ensure that patients receive the option to access all appropriate treatments could be to take the MDT into the outpatient setting. This takes the multidisciplinary care from not only treatment decision making, but also assessment. An MDT clinic may improve care pathways (with reduced waiting time) and allows the ‘benefit of the doubt’ to be given to patients that are of borderline fitness or are unsure. Just like patients need to be discussed at an MDT, patients may be reviewed by an MDT clinic to allow a patient-centred, MDT recommendation to be made regarding their care, and to be discussed with the patient to incorporate their views and preferences into the treatment decisions. The MDT clinic option is used in some settings but may not be generalisable because of the high cost of staffing these clinics.

Increasing subspecialities with an expanding curriculum has meant that there are increased pressures upon trainees to complete site-specific training. It is important that this is retained during training of future oncologists and that training is not diluted. Tumour site-specialist care has served us well, but it is increasingly apparent that future treatment algorithms will (and in some cases already are) based around molecular profiles of tumours, rather than their site of origin so ongoing review and innovation in team working will be needed.

There are unacceptable disparities in cancer care across United Kingdom. For example, in Northern Ireland, there has been delay in establishing the equivalent to the cancer drug fund system that operates elsewhere in the United Kingdom. This not only reduces access to patients to appropriate modern therapy but also diminishes the opportunities to conduct modern clinical trials where these drugs may be involved. These disparities are not acceptable to cancer patients or cancer professionals and the ACP will use its expertise and influence to ensure they are addressed.

### Actions

The ACP will undertake, with other organisations a series of initiatives to address the maintenance and development of multidisciplinary specialised care, and ensure equal access across United Kingdom:

1. Develop an early consultant senior clinical advisor or mentor system that will draw on professional support and advice for newly appointed consultants in their site-specialised role. We will establish a working group of senior and newly appointed consultants and trainees to develop this system. We currently propose for discussion that in the first three years of their consultant appointment every new consultant cancer physician will have a site-specialised senior clinical advisor or mentor, for each of the specific cancer sites for which they will provide multidisciplinary specialised care. The senior clinical advisor will be selected by the new consultant.
2. Develop further our established programme of Continuing Professional Development (CPD) and we completed a workshop on Precision Oncology in October 2015 and will publish the proceedings as an ACP textbook in 2016/17.
3. Use ACP expertise and influence to argue strongly for equal access to care and address disparities, including access to appropriate drugs across United Kingdom.

### Measures

- Notification of tumour site specialisations by consultants to the ACP.
- An agreed plan for the senior clinical advisor/mentor system in 2015.
- ACP workshop 2015 and publication on Precision Oncology 2016/17.

## Figures and Tables

**Table:** 

SUPPORTING CHAPTERS	CHAPTER AUTHORS	PAGE
**Delivering Excellent and Safe Medical Oncology**		
1. Multidisciplinary specialised care	Professor Johnathan Joffe (Huddersfield), Dr Adam Januszewski (London), Professor Peter Selby (Leeds)	17–20
2. Patient centred care	Professor Galina Velikova (Leeds), Dr Alex Mitchell (Leicester)	20–23
3. High-quality research and innovation	Dr Richard Baird (Cambridge), Professor John Chester (Cardiff), Professor Peter Johnson (Southampton)	24–25
4. An expert well-trained workforce	Dr Helena Earl (Cambridge), Professor David Cunningham (London), Dr Hannah Taylor (Bristol), Dr Sarah Payne (London), Dr Graham Dark (Newcastle), Alison Norton (London)	25–28
5. Development and growth of the discipline	Professor Johnathan Joffe (Huddersfield), Alison Norton (London), Dr Sarah Payne (London), Dr Adam Januszewski (London), Professor Peter Selby (Leeds)	28–32
6. Patient and public engagement	Mr Mark Flannagan (Beating Bowel Cancer), Professor Ian Banks (Belfast), Professor Johnathan Joffe (Huddersfield), Professor Peter Selby (Leeds)	32–33
**Addressing Challenges in the NHS**		
7. A comprehensive acute oncology service	Dr Ernie Marshall (Wirral), Dr Alison Young (Leeds), Dr Richard Griffiths (Wirral), Dr Tom Newsom-Davis (London), Dr Richard Osborne (Poole)	33–37
8. The care of older cancer patients	Dr Alistair Ring (London), Dr Janine Mansi (London), Dr Danielle Harari (London), Dr Tania Kalsi (London), Professor Peter Selby (Leeds)	37–42
**Innovation in Cancer Care**		
9. New ways to ensure the most timely access to best care	Professor Richard Neal (Bangor), Professor Peter Selby (Leeds), Professor Johnathan Joffe (Huddersfield)	42–45
10. Informatics and the delivery of new models of care	Dr Geoff Hall (Leeds), Dr Richard Griffiths (Wirral), Dr Adam Dangoor (Bristol)	45–48
11. Cancer survivors	Dr Jeff White (Glasgow), Professor Michael Hawkins (Birmingham), Dr Rod Skinner (Newcastle), Professor John Radford (Manchester), Professor Adam Glaser (Leeds), Dr Dan Stark (Leeds)	48–53
12. Precision oncology and genetics	Dr Ellen Copson (Southampton), Dr Zoe Kemp (London), Professor Diana Eccles (Southampton), Professor Peter Johnson (Southampton), Dr Emily Shaw (Southampton)	54–58

## References

[1] Sainsbury R, Haward B, Rider L (1995). Influence of clinician workload and patterns of treatment on survival from breast cancer. Lancet.

[2] Selby P, Gillis C, Haward R (1996). Benefits from specialised cancer care. Lancet.

[3] Mikeljevic JS, Haward RA, Johnston C (2003). Surgeon workload and survival from breast cancer. Br J Cancer.

[4] Downing A, Mikeljevic JS, Haward B (2007). Variation in the treatment of cervical cancer patients and the effect of consultant workload on survival: a population-based study. Eur J Cancer.

[5] Morris E, Quirke P, Thomas JD (2008). Unacceptable variation in abdominoperineal excision rates for rectal cancer: time to intervene?. Gut.

[6] National cancer peer review (NCPR) database (2010–2013). The cancer quality improvement network system; a web based database used to support the peer review process. http://www.cquins.nhs.uk.

[7] Survival estimates were provided by the Cancer Research UK Cancer Survival Group, London School of Hygiene and Tropical Medicine on request, 2011. http://www.lshtm.ac.uk/eph/ncde/cancersurvival.

[8] Autier P, Boniol M, La Vecchia C (2010). Disparities in breast cancer mortality trends between 30 European countries: retrospective trend analysis of WHO mortality database. BMJ.

[9] Autier P, Boniol M, Gavin A (2011). Breast cancer mortality in neighbouring European countries with different levels of screening but similar access to treatment: trend analysis of WHO mortality database. BMJ.

[10] National cancer action team (2010). The characteristics of an effective multidisciplinary team (MDT). http://webarchive.nationalarchives.gov.uk/20130513211237/http://www.ncat.nhs.uk/.

[11] Fleissig A, Jenkins V, Catt S (2006). Multidisciplinary teams in cancer care: are they effective in the UK?. Lancet Oncol.

[12] Borras JM, Albreht T, Audisio R (2014). Policy statement on multidisciplinary cancer care. Eur J Cancer.

[13] Haidet P, Fecile ML, West HF (2009). Reconsidering the team concept: educational implications for patient-centered cancer care. Patient Educ Couns.

[14] Wright FC, De Vito C, Langer B (2007). Multidisciplinary cancer conferences: a systematic review and development of practice standards. Eur J Cancer.

[15] Lamb BW, Brown KF, Nagpal K (2011). Quality of care management decisions by multidisciplinary cancer teams: a systematic review. Ann Surg Oncol.

[16] Lamb BW, Taylor C, Lamb JN (2013). Facilitators and barriers to team working and patient centeredness in multidisciplinary cancer teams: findings of a national study. Ann Surg Oncol.

[17] Dew K, Stubbe M, Signal L (2015). Cancer care decision making in multidisciplinary meetings Qual Health Res.

[18] Blazeby JM, Wilson L, Metcalfe C (2006). Analysis of clinical decision-making in multi-disciplinary cancer teams. Ann Oncol.

[19] Saini KS, Taylor C, Ramirez A-J (2012). Role of the multidisciplinary team in breast cancer management: results from a large international survey involving 39 countries. Ann Oncol.

[20] Hong NJL, Wright FC, Gagliardi AR (2010). Examining the potential relationship between multidisciplinary cancer care and patient survival: an international literature review. J Surg Oncol.

[21] Barry MJ, Edgman-Levitan S (2012). Shared decision making – the pinnacle of patient-centered care. N Engl J Med.

[22] Legare F, Stacey D, Turcotte S (2015). Interventions for improving the adoption of shared decision making by healthcare professionals. Cochrane Database Syst Rev.

[23] Taylor C, Finnegan-John J, Green J (2014). “No decision about me without me” in the context of cancer multidisciplinary team meetings: a qualitative interview study. BMC Health Serv Res.

# Brief report

**Published:** 2016

## 2. Patient centred care

**Professor Galina Velikova and Dr Alex Mitchell**

Sustain a strong, patient-centred focus which influences all aspects of practice throughout patients’ lifetimes with and after cancer, often using patient reported outcome measures.

All cancer care is patient-centred in the sense that benefits for patients and improving outcomes is the ultimate goal. However, in recent decades, it has been accepted that high-quality patient-centred care should explicitly extend beyond the appropriate and safe delivery of state-of-the art diagnosis and treatment towards a more inclusive approach. This includes involving patients and families in shaping cancer services, collecting feedback, which is then used to inform practice [1]. Patient and public involvement has driven changes positively and is now a recognised component of care planning, care delivery and research and innovation.

Patient-centred care takes account of the individual needs of patients and their carers, builds their views into the diagnostic and treatment processes and professionally and sympathetically presents the choices that are open to them between different treatments. This approach relies on effective and compassionate patient-professional communication to identify needs and on appropriately structured cancer services to respond. The importance of better communication with patients and families, to understand their priorities and preferences and include those in a shared decision-making process, has been recognised.

Cancer diagnosis and treatment brings significant emotional burden and distress to patients and their families. Detecting psychological morbidity and providing timely supportive care is a key goal of all cancer professionals, working in collaboration with psychooncology services and mental health professionals.

An essential component of patient-centred care is the ability to routinely measure patient-centred outcomes of care using patient-reported outcome measures (PROMs), such as measures of symptoms burden, psychological distress screening, impact on functioning and health-related quality of life, or satisfaction with care. PROMs are increasingly acknowledged as a valuable measure of the success of cancer treatments, in addition to objective measures of survival and cure rates.

These broad patient-centred approaches will remain central to the practice of medical oncology in coming years. ACP will continue to strongly support their wider implementation.

### Patient-professional communication and shared decision-making

A strong evidence-base exists supporting the value of advanced communication skills training using a learner-focused approach [2, 3]. The training leads to improvements in key communication behaviours related to information gathering and supportive skills. ACP acknowledges that basic training in communications skills is now an essential part of the undergraduate medical curriculum and our trainees enter oncology with significant skills in sensitive communication with vulnerable patients, breaking bad news and supporting distressed patients. However, there is an added value of advanced communication training using a learner-centred approach, helping cancer professionals to develop communication strategies in situations they personally find difficult and challenging. We know that training experienced senior oncologists has been beneficial [3].

In the past decade the cancer networks rolled out the oncology communication skills training programme, providing access for all members of the multidisciplinary cancer teams. Many oncology trainees and consultants benefited from these courses. However, with the reorganisation of the cancer networks, there is danger that these courses will not continue, or their number will reduce significantly limiting access for trainees. The ACP emphasises the need to continue to offer high-quality advanced communication skills training to oncology professionals of all professions.

### Psychosocial oncology and supportive care

Psychological and psychiatric complications of cancer are common in the early and late stages of cancer and in long term cancer survivors. Many patients with cancer report that they have unmet emotional, social, or spiritual (psychosocial) needs [4–6]. Untreated, mental health problems reduce quality of life and may adversely impair receipt of medical treatment for cancer [7]. There is now robust evidence base that psychosocial interventions (cognitive behavioural therapy, group therapy) are effective in reducing depression, improving quality of life, coping skills, and self-esteem [8, 9]. Delivering psychosocial interventions remains challenging. Potentially useful options include telephone key worker, web-based supportive care, and computer-based assessments/treatment. Peer support may be very powerful. Collaborative care is an interesting option (adopted from primary care) where there is clear input from mental health specialists [10].

Patients’ needs for psychological support may not be met for several reasons. Patients’ needs often are not recognised by professionals, who consequently may not offer support or referral. Services from which they might benefit may not be locally available to an adequate standard. Patients may not be willing or able to ask for help at the right time. Poor interprofessional communication and co-ordination can lead to suboptimal care.

Since 2004, NICE recommended that all patients with cancer undergo regular systematic psychological assessment at key points in their pathway and have access to an appropriate level of psychological intervention [11]. A four-level model of professional psychological assessment and intervention was endorsed. Professional psychological support at Levels 1 and 2 is provided by health professionals directly responsible for the care of people with cancer. More severe psychological distress (Levels 3 and 4) are managed by a variety of psychological specialists, including counsellors, mental health nurses, clinical and health psychologists, psychotherapists and liaison psychiatrists. It is also essential that professionals empower patients to recognise and with support self-manage their psychological needs.

Medical oncologists have an important role in the psychological care of patients, which includes responsibilities for identifying distress, referral to appropriate services, and prescription of antidepressant and other psychotropic medication. Where specific psychooncology services are not available in the hospitals, medical oncologists should identify other existing similar services locally (including in primary care, hospices and the community) and develop structured referral pathways for patients with cancer.

ACP endorses continued implementation of NICE recommended model for psychological support services, with emphasis on the following:

- communication skills training;
- the wider use of formal screening for distress (electronic PROMs and health informatics approaches);
- training in the management of low levels of distress for appropriate members of the multi-disciplinary team;
- close collaborations with colleagues in clinical psychology, psychooncology, and psychiatry.

### Patient-reported outcomes

The challenges of reliably and validly measuring the quality of patient experience and of patient outcomes have been substantial. Many innovative approaches using scientifically valid, psychometric approaches, have been developed and deployed in clinical research and the practice of oncology. UK medical oncologists have contributed substantially, nationally and internationally, to the development of the appropriate measurement methods, commonly now referred to as patient reported outcome measures (PROMs) and to their evaluation in clinical trials and in oncology practice [12, 13]. This work is being extended to encompass large-scale surveys of cancer patient populations [14, 15]. However, it is the time for feedback from patients and their families to become more rigorous, routine, integrated in health care and used to inform practice, not merely collated for research [1]. Electronic methods for collecting PROMs (ePROMs) have the potential to facilitate patient-centred care during and after treatment (follow-up of cancer survivors): they are convenient for patients, increase data accuracy, reduce long-term costs and provide large data sets detailing patient experiences. The data can be prospectively analysed in a systematic way to evaluate services and build the knowledge-base on subjective effects of cancer therapies.

The National Information Board (NIB) have developed an action framework for the use of data and technology to transform outcomes for patients, highlighting that patients/public will use technology for health reasons [16].

ePROMs are being implemented to remotely monitor patients symptoms or adverse events and to screen for psychological morbidity [17–20]. However, for this approach, to become truly useful for patient care, it is essential to integrate ePROMs with the electronic patient records (EPR) to inform care and provide rapid access to specialist clinicians [21, 22]. The future of patient-centred care will rely on health informatics, will promote intrinsic patient involvement via patient portals, integrating ePROMs with EPR to routinely monitor toxicity, inform individual care, collect comparative effectiveness data on treatments and service evaluation. The great potential of ePROMs is recognised but key challenges remain, including developing specific e-platforms, their interoperability and integration with EPR and clinical trials databases, and developing innovative care models that include ePROMs to monitor symptoms and toxicity with clinical algorithms leading to appropriate management. These innovative approaches require more pragmatic research leading directly to practical use. However, successful implementation will ultimately depend on the acceptance by cancer professionals. To this end, ACP will support training strategies for the multidisciplinary cancer teams to learn, understand and use ePROMs. A review of existing ePROMs systems for toxicity monitoring is also recommended.

### Actions

The ACP will contribute to maintaining and developing patient-centred approach in a number of ways:

1. Continuing to incorporate substantial training and CPD in advanced communication skills. Collect information on available advanced communication skills training courses.
2. Promote the inclusion of psychosocial aspects of health care in all cancer services, which we lead and/or provide. Close collaborations with colleagues in psychiatry, clinical psychology, and general practice will remain important in multiprofessional practice in oncology.
3. ACP workshop on patient-centred and integrated cancer care in October 2016 with a problem-solving textbook in 2017.
4. Workshop/project to review and collate information on existing electronic systems for patient reporting of side effects, in order to develop recommendations for best practice and support wider implementation.

### Measures

- Eighty percent of trainees and consultants have completed advanced communication skills training within 5 years.
- Completion of ACP workshop and problem-solving textbook on psychosocial aspects of cancer xare, 2016/2017.
- Completion of review of electronic data capture systems.

## References

[1] Coulter A, Locock L, Ziebland S (2014). Collecting data on patient experience is not enough: they must be used. BMJ.

[2] Moore PM, Rivera Mercado S, Grez Artigues M (2013). Communication skills training for healthcare professionals working with people who have cancer. Cochrane Database Syst Rev.

[3] Fallowfield L, Jenkins V, Farewell V (2002). Efficacy of a cancer research UK communication skills training model for oncologists: a randomised controlled trial. Lancet.

[4] James DH, Jane MY, Melanie AP (2009). What are the unmet supportive care needs of people with cancer? a systematic review. Supportive Care In Cancer.

[5] Armes J, Crowe M, Colbourne L (2009). Patients’ supportive care needs beyond the end of cancer treatment: a prospective, longitudinal survey. J Clin Oncol.

[6] Mitchell AJ, Ferguson DW, Gill J (2013). Depression and anxiety in long-term cancer survivors compared with spouses and healthy controls: a systematic review and meta-analysis. Lancet Oncol.

[7] Goodwin JS, Zhang DD, Ostir GV (2004). Effect of depression on diagnosis, treatment, and survival of older women with breast cancer. J Am Geriatr Soc.

[8] Jacobsen PB, Donovan KA, Swaine ZN, Chang AE, Ganz PA, Hayes DF (2006). Management of anxiety and depression in adult cancer patients: toward an evidence-based approach.

[9] Williams S, Dale J (2006). The effectiveness of treatment for depression/depressive symptoms in adults with cancer: a systematic review. Br J Cancer.

[10] Sharpe M, Walker J, Hansen CH, SMaRT (Symptom Management Research Trials) Oncology-2 Team (2014). Integrated collaborative care for comorbid major depression in patients with cancer (SMaRT Oncology-2): a multicentre randomised controlled effectiveness trial. Lancet.

[11] (2004). National institute for clinical excellence: improving supportive and palliative care for adults with cancer: the manual.

[12] Velikova G, Booth L, Smith AB (2004). Measuring quality of life in routine oncology practice improves communication and patient well-being: a randomized controlled trial. J Clin Oncol.

[13] Kotronoulas G, Kearney N, Maguire R (2014). What is the value of the routine use of patient-reported outcome measures toward improvement of patient outcomes, processes of care, and health service outcomes in cancer care? A systematic review of controlled trials. J Clin Oncol.

[14] Ashley L, Jones H, Thomas J (2013). Integrating patient reported outcomes with clinical cancer registry data: a feasibility study of the electronic patient-reported outcomes from cancer survivors (ePOCS) system. J Med Internet Res.

[15] Glaser AW, Fraser LK, Corner J (2013). Patient-reported outcomes of cancer survivors in England 1-5 years after diagnosis: a cross-sectional survey. BMJ Open.

[16] National Information Board (2014). Personalised Health and Care 2020: using data and technology to transform outcomes for patients and citizens a framework for action.

[17] Judson TJ, Bennett AV, Rogak LJ (2013). Feasibility of long-term patient self-reporting of toxicities from home via the internet during routine chemotherapy. J Clin Oncol.

[18] Basch E, Reeve BB, Mitchell SA (2014). Development of the national cancer institute’s patient-reported outcomes version of the common terminology criteria for adverse events (PRO-CTCAE). J Natl Cancer Inst.

[19] Kearney N, McCann L, Norrie J (2009). Evaluation of a mobile phone-based, advanced symptom management system (ASyMS) in the management of chemotherapy-related toxicity. Support Care Cancer.

[20] Carlson LE, Groff SL, Maciejewski O (2010). Screening for distress in lung and breast cancer outpatients: a randomized controlled trial. J Clin Oncol.

[21] Jensen RE, Snyder CF, Abernethy AP (2014). Review of electronic patient-reported outcomes systems used in cancer clinical care. J Oncol Pract.

[22] Ziegler L, Harley C, Holch P (2012). Towards safer delivery and monitoring of cancer treatments Electronic patient self-reporting of adverse-events: patient information and advice (eRAPID). Psycho-Oncol.

# Brief report

**Published:** 2016

## 3. High-quality research and innovation

**Dr Richard Baird, Professor John Chester, and Professor Peter Johnson**

Continue our commitment to conduct high-quality research and innovation to answer key questions that will determine optimal patient management and to ensure the translation of new knowledge from the laboratory to the clinic and from clinical evidence into clinical practice. We will commit to sustaining the role of UK medical oncology as a research-intensive discipline, at the forefront of biomedical and health research in the United Kingdom and internationally.

Research and innovation on new drugs and biological therapies, and their optimum usage via ‘precision or stratified medicine’, are central to the research activities of Association of Cancer Physicians members. Many medical oncologists conduct laboratory and/or clinical trials research into the causes and cure of cancer as part of their portfolio of professional roles. Medical oncologists are and will continue to be, at the forefront of research and innovation into the treatment of cancers and their delivery into routine patient care. Many develop and coordinate clinical trials and all participate in recruitment of patients to clinical trials, as indicated in the RCP publication ‘Physicians working with patients’.

In addition, medical oncologists, as opinion-leaders within the wider cancer care and cancer research community, have significant opportunities to influence and support work on prevention, screening, early diagnosis and survivorship, and to inform and lobby for improved screening and lifestyle changes.

Clinical trials and research into optimal patient care, including applied health research and patient-centred approaches, are vital and will remain part of the research and innovation commitments of the Association of Cancer Physicians. In addition, many medical oncologists have additional research sessions dedicated to clinical trials and/or have leadership roles to develop clinical trial and other research infrastructures in their institutions. High-quality clinical and translational research is not possible without such infrastructure, and in the globally competitive field of clinical cancer research, it is increasingly important for investigators to meet targets for trial recruitment numbers and time-lines.

We will work in close partnership with the organisations that lead on cancer research. For example, we note the recent research strategy developed by Cancer Research UK, [1] and its headlines:

- Support the earlier diagnosis of cancer.
- Increase research in lung, pancreatic, oesophageal cancers and brain tumours.
- Remain focussed on rare cancers that receive less proportional research funding, and also the rare subtypes of commoner cancers that need specific research and management approaches.
- To understand better what causes and drives cancer.
- Increase prevention research and tobacco control.
- Discovery and development of new drugs, including biological therapies.
- Optimise the chance of survival for every individual, through precision medicine approaches.

Within these broad areas of activity medical oncologists will have substantial portfolios and contributions in most topics, with perhaps currently especially:

- Novel targeted systemic therapies.
- Biological and immunological therapies.
- Increasing to precision of treatment to improve therapies effectiveness and reduce toxicity, employing novel biomarkers and imaging strategies.

Many medical oncologists work in universities and research institutes. In the latest RCP census (2012), 31.2% of medical oncologists are employed wholly or partly by academic institutions. Just 57% are employed solely by the NHS, the lowest figure for any medical speciality with the exception of clinical pharmacology and metabolic medicine (both of which are numerically much smaller specialties – each having only 6 consultants in the census). Academic medical oncologists may be employed as professional researchers who run laboratories, clinical trials or applied health research portfolios. Sustaining a pipeline of such excellent academic medical oncologists is a key priority for our specialty.

The contribution to research and innovation of medical oncologists will not rest solely with those working in universities and related organisations, however. Participation in clinical trials is expected to form part of every medical oncologist’s job plan, included as direct clinical care (DCC) [2]. All medical oncologists must also be committed to and involved in the development of care and innovation to improve outcomes for patients and this should be recognised in job plans. This will involve contributing to clinical and applied health research, linking with laboratory researchers to promote translational research and a constant quest for innovation to improve outcomes within their own services and more widely. Clinical trials are the key to improved cancer outcomes. They are an essential, cost-effective method of driving up care quality and support is required at all levels of the NHS in all the countries of the United Kingdom. There are substantial differences in the clinical research opportunities for oncologists and cancer patients in Cancer Centres in Teaching Hospitals and those in District General Hospitals (DGH). Treatment options and therefore trials options may be more limited in DGHs. Clinical trials portfolios should include studies which allow important research questions to be answered by research conducted in DGH oncology services.

### Actions

1. A new ACP executive academic lead
2. Increased contributions to national cancer research meetings.
3. All members contribute to clinical trials which are appropriate to their institutions and the level of team support.

### Measures

- Research leadership roles
  - locally (e.g. local research lead for tumour type, trials unit director)
  - regional (e.g. regional research lead for tumour type)
  - national (e.g. Membership of funding committees, CSGs, etc.)
- Clinical trial recruitment metrics can provide useful information such as:
  - number of open trials on which a medical oncologist is CI or PI
  - total number of patients recruited to clinical trials

Although we must remain mindful of the goals of high-quality research remain, to improve clinical practice and patients survival and quality of life, not just to meet targets.

- Growth in academic trainees and academic consultant numbers

## References

[1] CR-UK Strategy. http://www.cancerresearchuk.org/sites/default/files/cruk_research_strategy.pdf.

[2] (2013).

# Brief report

**Published:** 2016

## 4. An expert well-trained workforce

**Dr Helena Earl, Professor David Cunningham, Dr Hannah Taylor, Dr Sarah Payne, Dr Graham Dark, and Alison Norton**

Produce an expert workforce through a well-planned training programme to address knowledge, skills, and competency, with excellent Continuing Professional Development and a robust and supportive trainee and young consultant advisory or mentor system.

### (a) Education, training, and CPD

We must ensure that the skills of oncologists remain up to date and relevant, hence supporting the provision of best care for patients. The current Medical Oncology specialist trainee curriculum lasts 4 years and was developed by the Specialty Advisory Committee for Medical Oncology with representation from the Association of Cancer Physicians under the direction of the Joint Royal Colleges of Physicians Training Board (JRCPTB). This builds on the skills acquired during core medical training to develop the specific competencies required to practise independently as a Medical Oncologist.

The curriculum is reviewed regularly to ensure the skill set of oncologists is relevant to the changing demands on the Medical Oncology service. As discussed, the current pressures include the development of acute oncology [1, 2] the importance of improving oncological care pathways for the management of the elderly population and the need to stay up to date with regard to the management of acute medical problems pertinent to the oncology patient and to stay on top of the rapid expansion of new knowledge in oncology.

There is currently only limited time dedicated to non-surgical oncology in undergraduate education (WHO stipulate only 2 weeks). Additionally, less than 60% of postgraduate trainees at FY/CMT currently have the chance to include oncology in their medical rotations [2, 3]. The paucity of oncology exposure clearly endorses the importance of the opportunity to use acute oncology as an educational tool for primary, secondary, and tertiary care. Education and training is also a very important means of facilitating collaborative working with other medical specialties, and with primary care and sustaining and strengthening the MDT.

A combination of workshops, publications and continuing medical education plans (e.g. Acute Oncology Services Study day 2012 [1], Cancer Care in Elderly Patients Study Day 2014 [4]) have been and are being put in place aimed at oncology trainees and consultants, nurses and allied health professionals. The success of these programmes relies on the close collaboration with allied medical specialties. To date, links are being established between geriatrics and oncology using innovative service developments. Furthermore, novel liaisons between Medical Oncology and Clinical Genetics are being established through pilot studies supporting more direct involvement of the Medical Oncology team in assessing and consenting patients for genetic screening and testing. This is likely to enhance both patient experience and training and should represent a model for reviewing the development of other oncology subspecialist services.

Within the training programme, it is also important we highlight the commitment to training in research methodology, audit, management and education and opportunities to develop these skills in these areas. Research is and must be a key component of training and development for all trainees and then for all consultants.

It is fundamental that all cancer physicians continue to be trained to a high standard to provide care for patients with cancer across their whole journey and to be able to provide acute oncological care in an unselected way. To support this, training may need to be broadened, for example, a more thorough grounding in the principles of radiotherapy, palliative care, and clinical genetics. They must orchestrate the care of patients effectively, working closely with the many other professions and disciplines who contribute to the best patient outcomes, and colleagues in primary care, in particular. This is already a key theme in training and is likely to become even more so, in future.

The need to train medical oncologists to the highest standards to deliver excellence as rounded oncologists giving safe and effective treatment is paramount, but with a training period of 4 years, it is already becoming a challenge to cover all the new innovations in medical oncology. Trainees usually enter speciality training with little experience of outpatient oncology, such as chemotherapy prescribing and the side effects of treatment, management strategies, and assessing fitness for treatment which make up the bulk of the work. Oncology treatments are also developing at a rapid rate and trainees must keep up with these developments across all tumour sites. Add in the changes in personalised medicine and genetics, and the difficulties in adequately covering all of this in a 4-year training period become apparent, and the need for strong support for ongoing learning as consultants becomes ever more important.

Current UK trainees rank training programmes highly (Biannual Trainee Survey 2013 [5]). Trainees are regularly engaged in the continuing improvement and review of curriculum. This continuing engagement is fundamental to ensure that training remains relevant, current and of a high standard thus maintaining an excellent senior workforce providing excellent patient care when a CCT must equate to being competent to practice independently.

### (b) Pressure on serviced provision and general medical training

Clearly, regulation of the balance between training and service provision is becoming more complex with the reconfiguration of services. As a profession, we believe that trainees and consultants must develop and retain general medical skills to cover the needs of a very large patient population with cancer across all of the patient pathway. This is central to continuing to improve cancer outcomes. This has many facets that should be reflected in training programmes and in continuing professional development and improvement. We accept that strengthening the training of cancer physicians in general medical aspects of their care, including the ability to provide support to unselected populations of cancer patients at the point of diagnosis, management and acute complications and end-of-life care, could be achieved by some further training in general internal medicine as part of the training programme. This is always going to create pressures and tensions within crowded training programmes that are time and resource-limited.

As physicians we are mindful of the pressures presented to all of our colleagues by acute and unselected medical ‘take’. The multiple morbidities experienced by elderly patients will increase these pressures in future. Our training in acute oncology and our contribution to the acute access of patients with cancer to optimal care and our planned strengthening of care for older patients with cancer, is a substantial contribution to acute medicine. We have chosen to develop these additional skills and accept these responsibilities. Ideally, this would involve innovative liaison with relevant medical specialties to ensure optimal direction of patient care pathways and to enhance training experience.

It has been highlighted by trainees that attendance at formal teaching sessions is often affected by conflict with clinical commitments [6]. This is an area of which the ACP is mindful. Currently, the annual ARCP review ensures that training objectives are met satisfactorily and this is a process that will obviously continue. To complement this, regular trainee surveys completed by the GMC (annual) and ACP (biannual) and participation in ACP/SAC committee meetings currently ensure that there is always a voice from the grass roots to help feedback on how training is really going.

Consultant cancer physicians will be grounded in general medicine and deploy those skills in the care of cancer patients but they should not undertake acute unselected general medical take. That would be a poor use of their skills in cancer care, reduce their ability to deliver excellent specialised cancer care and will result in poorcancer patient outcomes and an over-pressurised and ineffective profession. We do believe, however, that the contribution of cancer physicians to acute oncology will make a substantial and helpful and cost-effective contribution to the management of all cancer patients when they are in contact with cancer services, including the ageing cancer patient population.

### Actions

- A working party on workforce planning and training numbers jointly with clinical oncologists.
- Acute oncology service developments, training and CPD (published text 2014) [1].
- Training and CPD to address the problems of older cancer patients (published text 2016) [6], precision oncology and cancer genetics (Workshop October 2015).

### Measures

- Biannual survey feedback.
- Annual appraisal of training outcomes.

## References

[1] Marshall E, Young A, Clark P, Selby P (2014). Problem solving in acute oncology.

[2] Payne S, Burke D, Mansi J, Jones A, Norton A (2013). Discordance between cancer prevalence and training: a need for an increase in oncology education. Clin Med.

[3] Cave J, Woolf K, Dacre J, Potts HW, Jones A (2007). Medical student teaching in the UK: how well are newly qualified doctors prepared for their role caring for patients with cancer in hospital? Br J Cancer.

[4] Ring A, Harari D, Kalsi T, Mansi J, Selby P (2016). Problem Solving in Older Cancer Patients.

[5] (2013).

[6] Ring A, Harari D, Kalsi T, Mansi T, Selby P (2016). Problem solving in older cancer patients.

# Brief report

**Published:** 2016

## 5. Development and growth of the discipline

**Professor Johnathan Joffe, Alison Norton, Dr Sarah Payne, Dr Adam Januszewski, and Professor Peter Selby**

Ensure that the growth of our discipline matches the needs of our patients and future cancer services, with the flexibility to adapt to changing needs/demographics.

We believe that medical oncology consultant numbers need to grow to 1.0 FTE per 100,000 UK residents by 2020 and to 1.5 FTE per 100,000 UK residents as soon as possible after 2020, and be evenly distributed across the UK, which means the number of consultants will grow to a total of over 900 full time equivalents. Where medical oncologists take on roles in oncology service developments in addition to delivering SACT, then specific additional funded protected time and support teams will be essential.

In the last decade, there has been steady growth in consultant medical oncology numbers [1] (Figures 1–4) by some 20 posts per year (varying between 2% and 12% pa), although the posts are unevenly spread across United Kingdom.

The ageing population is resulting in some 2–3% pa increase in new cancer cases. It is difficult to see that the increasing number and complexity of cases can be managed without a radical increase in consultant posts with attention given to a more even national distribution. It needs to be acknowledged that a high proportion of our consultants have only part-time clinical commitments (due to academic research commitments, thus comparison with some of the larger specialties in terms of numbers of consultants per head of population, may be misleading and we should focus our data on whole time equivalents wherever possible.

This expansion has continued in 2012–2013 and medical oncology remains a significant growth area in medicine compared to other specialties.

The Royal College of Physicians estimate that to deliver a robust Acute Oncology service across the country, a significant increase in medical oncologists is required. The need to safely deliver modern care and the growth of new areas of activity must bring the number of medical oncologists per head of population to considerably more than 1:100,000, and this number is currently only provided in North West London [2]. In 2011 (revised in 2013), the RCP recommended a requirement of a minimum of 2.75 FTE posts per 200,000–250,000 population (representing 705–881 posts for United Kingdom, at 2013 population estimates). However, the specialty advisory committee (SAC) and the Association of Cancer Physicians has recently submitted a revised estimate of 1.5 FTE per 100,000 population (representing 962 posts for United Kingdom). The European Society of Medical Oncology coordinates the development of medical oncology in Europe [3] and a recent publication [4] has noted that among 12 European countries which provided data on consultant medical oncology numbers, the United Kingdom has the highest ratio of patients with cancer to medical oncologists, but the fastest rate of growth in the specialty.

The ICT reviewed the workforce implications of its new plan and of the current state of workforce planning for medical oncology and related oncology disciplines. It noted and endorsed our core goal of one consultant medical oncologist per 100,000 population before 2020. However, it also recognised the need to go beyond this and the report comments that the need is likely to grow further to around 1 FTE medical oncologist per 80,000 population beyond 2020.

There are opportunities to mobilise efficiencies to reduce pressure of consultant medical oncologists. When, as ultimately inevitable, comprehensive and effective health informatics is introduced into the NHS the impact on workload will be helpful. This will improve access, reduce patient administration and improve safety and quality of care. Similarly, the availability of remote patient monitoring and internet monitoring for complications of cancer or of its treatment may reduce the requirement for face-to-face contact between cancer patients and oncologists.

Increasingly, new targeted therapies are providing survival benefits for patients that were not imagined 20 or 30 years ago. Many patients now live with active cancer that is controlled by systemic therapies, such as trastuzumab, in breast cancer or gefitinib in lung cancer. These patients require ongoing monitoring of side effects and effectiveness of treatment that may extend over many years. In the next 20–30 years, there will be many more new targeted and scientifically based treatments that will turn many cancers in to long-term conditions. Even with anticipated increases in medical oncology manpower, the burden of care will be extremely challenging to manage.

Clinical oncology colleagues deliver substantial local and systemic cancer care, usually working in MDTs with medical oncologists. Workforce planning between these two specialties needs to be collaborative http://www.rcr.ac.uk [5, 6]. There are opportunities to develop the skills of colleagues in nursing and allied health professions to address some of the challenges of cancer care.

The growth of the specialty requires adequate training numbers, new consultant posts and the appropriate facilities and support and the retention of existing consultants for whom overseas posts, private sector work and reductions in stress and job pressure through retirement or part time working, may be attractive.

The ACP has considered the recommendations of the ICT for 1 FTE consultant medical oncologist per 80,000 people, that is, 1.2 FTE per 100,000 people, RCP, the European data, the growth achieved in the last decade and will strongly advocate two stages of development:

- The creation of 20–25 new consultant full-time equivalent posts per year from 2016 to 2020, approximately 125 new posts (FTE) added to the existing 450 posts. This will bring us close to the goal of 600 total FTE consultant posts and is feasible given previous growth, with an energetic national effort in training, recruitment, and retention.
- Developing a strategic plan to increase the total number of posts to 1.5 FTE consultants per 100,000 UK residents will require a radical increase in training numbers and potentially recruitment from overseas. We will argue strongly that this is needed and that this number must be achieved substantially by growth in medical oncology. Some of the needs may be met by growth in the number of clinical oncologists; some by innovation in the contributions of other professions and all of these approaches must be energetically pursued.

### Actions

- Establish a joint working party on workforce planning with the Royal College of Radiologists (Commitment 4). We will establish, with colleagues from clinical oncology, the National requirement for WTE consultants engaged in delivery of systemic therapies for cancer, now and for the future. We will be cognisant of the need to expand the workforce in radiation oncology also.
- We currently believe that this will require at least 1.0 FTE consultant medical oncologists per 100,000 UK residents by 2020 to ensure safe and effective delivery of systemic therapy and to improve and deliver many other aspects of excellent cancer care. This is likely to need to increase to 1.5 FTE consultants per 100,000 people as soon as that is possible.
- Ensure that medical oncologists who contribute to broader aspects of care (eg acute oncology, survivorship) have sufficient protected time for this work, in addition to work to deliver systemic anti-cancer treatment.

### Measures

- Consultant numbers and their distribution across United Kingdom.
- Regular review of available posts and unfilled posts.

## Figures and Tables

**Figure 1.** Data provided by the Royal College of Physicians shows a steady increase in the total number of consultant medical oncologists in the United Kingdom growing from just over 150 in 2002 to over 400 in 2013. Reproduced with permission. (Federation of the Royal Colleges of Physicians of the UK. *Census of consultant physicians and higher specialty trainees in the UK: specialty reports, 2013–14*. London: Royal College of Physicians, 2015: 155 and 157.)

**Figure 2.** Data provided by the Royal College of Physicians shows the distribution of medical oncologists between the countries of the United Kingdom and the continued expansion between 2012/13 and 2013/14. The rate of expansion is faster than that across all medical specialties. Reproduced with permission. (Federation of the Royal Colleges of Physicians of the UK. *Census of consultant physicians and higher specialty trainees in the UK: specialty reports, 2013–14*. London: Royal College of Physicians, 2015: 155 and 157.)

**Figure 3.** Data provided by the Royal College of Physicians shows an uneven distribution of medical oncologists across the United Kingdom. Reproduced with permission. (Federation of the Royal Colleges of Physicians of the UK. *Census of consultant physicians and higher specialty trainees in the UK: specialty reports, 2013–14*. London: Royal College of Physicians, 2015: 155 and 157.)

**Figure 4.** Data provided by the Royal College of Physicians shows that medical oncology is growing rapidly and is one of the fasting growing sub-specialties of Medicine. Reproduced with permission. (Federation of the Royal Colleges of Physicians of the UK. *Census of consultant physicians and higher specialty trainees in the UK, 2013–14: data and commentary*. London: Royal College of Physicians, 2015: 8.)

## References

[1] (2014). Federation of the Royal Colleges of Physicians of the UK; Census of consultant physicians and medical registrars in the UK, (2012): data and commentary.

[2] (2013). Consultant physicians working with patients.

[3] http://www.esmo.org/.

[4] de Azambuja E, Ameye L, Paesmans M (2014). The landscape of medical oncology in Europe by 2020. Ann Oncol.

[5] (2014). Clinical oncology – the future shape of the specialty Clinical oncology workforce: the case for expansion. http://www.rcr.ac.uk.

[6] Lievens Y, Defourny N, Coffey M (2014). Radiotherapy staffing in the European countries: final results from the ESTRO-HERO survey. Radiother Oncol.

# Brief report

**Published:** 2016

## 6. Patient and public engagement

**Mr Mark Flannagan, Professor Ian Banks, Professor Johnathan Joffe, and Professor Peter Selby**

Increasing patient engagement is central to the work of the ACP and the delivery of cancer care.

Oncology practice has been at the forefront of patient engagement, both in the development of shared decision taking in clinical practice and in the involvement of patients and patient representatives in the strategic planning of cancer care and of cancer research. The ACP wishes to sustain and strengthen this approach in coming years. Two patients were appointed to contribute to the drafting of this strategy and will become full members of the ACP Executive.

The ACP agrees with the NHS Confederation [1, 2] that:

Patient and public engagement (PPE) needs to be integral to everything that NHS organisations do, both as commissioners and providers of services. While techniques and approaches will differ depending on the particular circumstances and audiences, there are common principles and characteristics that underpin good engagement. Good engagement is focused on culture rather than structures or techniques; integral to all activity; strategic, clear and coordinated; open and transparent; well resourced and supported; inclusive and representative; flexible; collaborative and builds partnerships; sustained; outcomes based and focused on improvement.

In addition to our professional commitment to PPE, we will work in a new legal framework. The Health and Social Care Act 2012 has updated the NHS approach to PPE in England from that set out in earlier Acts. There is a legal duty to involve and consult patients in the planning and developing of new services, which was brought into law, through the Act. The Care Quality Commission requires providers to assess the views and experience of their service users.

Analyses of PPE show considerable progress, but there remains a great deal to be done to demonstrate the impact of PPE across healthcare systems [3, 4]. The impact of PPE in cancer research and in the wider domain of clinical research is increasingly recognised [4] and is strongly supported by the INVOLVE organisation in the United Kingdom [5]. Clinical Commissioning Groups and Commissioners in NHS England are required to enable patients and carers to participate in planning, managing, and making decisions about their care and treatment through the services they commission. They are also expected to enable the effective participation of the public in the commissioning process itself so that the services provided reflect the needs of local people [6, 7]. Leading UK cancer care organisations have now begun to systematically identify projects which have involved patients and the public [8]. Leading European cancer organisations have introduced patient representation at the most senior level [9, 10].

The work of ACP on PPE will be developed by a new group. We anticipate initial consultation with patient advocacy charities and individual patient surveys. This wider consultation will initially focus on the content and development of this strategy but should also generate new initiatives. We are mindful that collaboration with other leading oncology organisations may be the most efficient way to elicit increased patient inputs.

### Actions

- Develop a new ACP patient and public engagement (PPE) group and establish support and training for PPE in the work of the ACP.
- Through this group, to consult widely with patient advocacy organisations and individual patients, on their priorities for improved care.

### Measures

- The appointment and involvement of patients both for the drafting group for the strategy and for future ACP executive meetings.
- Establishment of the ACP PPE group in 2015/16.

## References

[1] www.nhsconfed.org.

[2] (2010).

[3] Mockford C, Staniszewska S, Griffiths F (2012). The impact of patient and public involvement on UK NHS health care: a systematic review. Int J Qual Health Care.

[4] Stewart D, Wilson R, Selby P (2011). Patient and public involvement. Ann Oncol.

[5] www.invo.org.uk.

[6] Légaré F, Boivin A, van der Weijden T (2011). Patient and public involvement in clinical practice guidelines: a knowledge synthesis of existing programs. Med Decis Making.

[7] Burns KK, Bellows M, Eigenseher C (2014). ‘Practical’ resources to support patient and family engagement in healthcare decisions: a scoping review. BMC Health Serv Res.

[8] www.christie.nhs.uk/about-the-christie/patient-public-involvement/examples-of-projects.aspx.

[9] http://www.ecco-org.eu.

[10] http://www.esmo.org.

# Brief report

**Published:** 2016

## 7. A comprehensive acute oncology service

**Dr Ernie Marshall, Dr Alison Young, Dr Richard Griffiths, Dr Tom Newsom-Davis, and Dr Richard Osborne**

Complete the Development of a Comprehensive Acute Oncology (AO) Service Nationwide, including Provision of Timely and Dedicated Care for Patients Whose Primary Cancer Site is Unknown.

There is a need for prompt access to acute oncological care for all patients to manage complications. Treatments must be provided through direct access, in a timely way, to specialists fully trained to cover all of the acute challenges. This contribution to the needs of patients becoming acutely unwell in the community or within the healthcare system, will make a substantial contribution to the need for improvements in acute medical services and probably accommodate some 5–10% of all acute ‘take’ patients who would otherwise be a challenge faced by acute unselected medical take services. Medical oncologists will provide acute care for cancer patients. This may be direct such as the provision of an ‘on take’ system for acutely unwell patients who are known to have cancer and undergoing management for this. Equally, medical oncologists will support other professions, disciplines, and specialties in their care for cancer patients, such as in primary care, general medicine and surgery, or nursing. For instance, acute oncology nurse practitioners are playing an increasing part in AO.

The National Audit Office Hospital Episode Statistics estimate that the number of patients receiving systemic anticancer chemotherapy (SACT) has been increasing year on year since 2001/02 (updated data), accounting for £1 billion expenditure annually. The National Confidential Enquiry into Patient Outcome and Death (NCEPOD) [1] published in 2008, evaluated the quality and safety of care for patients who died within 30 days of receiving SACT. The enquiry was set up especially to understand precisely the care pathways for this group of sick cancer patients. The care was deemed to be acceptable in only 35% of patients. In the 49% of patients where care was less than optimal, factors relating to both the organisation of emergency care as well as the specific care delivered by each institution were identified. The National Chemotherapy Advisory Group (NCAG) [2] was formed to address how care should be delivered, not only to improve the outcome of the sick cancer patient, but to also address key issues in the organisation of care to improve the patient experience.

The development of an Acute Oncology Service (AOS) in every NHS Hospital with an emergency department was a key recommendation of the NCAG report [2]. It described an AOS as one that brings together the expertise from oncology disciplines, emergency medicine, general medicine, and general surgery to ensure the rapid identification and prompt management of all patients who present with severe complications following chemotherapy or as a consequence of their cancer. Uniquely, it also described the management of patients who present as emergencies with previously undiagnosed cancer as a key responsibility of an AOS. These groups of patients, who present via acute medical departments with a constellation of symptoms and are subsequently found to have cancer, represent 22% of all new cancers diagnosed each year in England, with lung, pancreas, and brain malignant tumours forming the largest group. Data collected by the National Cancer Intelligence Network (NCIN) have shown that, apart from acute leukaemia, the relative survival for all patients diagnosed via the emergency route is worse than for those diagnosed by non-emergency services. Emergency presentation patients are usually of poor performance status, often elderly, and have multiple comorbidities. They are less likely to receive a histopathological diagnosis of cancer, to see a specialist in their tumour type, or to receive treatment. It is clear this group of patients need properly co-ordinated pathways with early oncology and palliative care input to ensure appropriate care is given.

We expect that the majority of medical oncologists will contribute to acute oncology services as part of their job plan. Some will take a special interest in the development of AO services and their leadership and will have clinical interests in AO-related areas such as cancer of unknown primary. Models will vary between hospitals and networks with varying inputs from medical and clinical oncology and general internal medicine and primary care. However, irrespective of local hospital or network solutions, acute oncology is underpinned by a number of core principles that promote education, awareness, and early access to specialist oncology teams. In these models, early specialist review must be combined with strong leadership and innovative service developments that will improve the safety and quality of emergency cancer care. The number and type of AO emergency admissions is highly dependent on local service configuration. This reflects the role of an individual hospital trust as an acute district general hospital, a fully integrated cancer centre or a standalone cancer centre that lacks acute medical and surgical support.

Data on acute oncology patterns and workload remain sparse. In 2006/2007, there were 273,000 emergency admissions with a diagnosis of cancer, representing a 30% increase from 1997/1998. This is roughly equivalent to 750 emergency admissions each day across England, so that a typical trust may have five emergency admissions with cancer per day (two under medicine, one under general surgery, one under oncology/haematology and one under ‘other’). Unplanned cancer admissions may happen several times for the same patient. Average length of stay for inpatient cancer admissions between regions varied from 5.1 to 10.1 days in 2008/2009. If every region had the same length of stay as the average in regions in the best performing quartile, even with no reduction in admissions, 566,000 bed days could be saved; equivalent to £113 million each year. The development of data on the impact of AO is essential for winning further support.

As a discipline, we responded with a strong commitment to the development of acute oncology [3, 4] and will continue to develop this broad contribution to acute medicine which involves 5–10% of any unselected acute intake into a hospital. In the coming years, medical oncology (MO) trainees will be required to develop a comprehensive understanding of acute oncology skills and service requirements.

The development of AO services remains patchy across England with many areas of innovation but also areas that are totally non-compliant. This is highlighted by national peer review (2012/2013 report [5]) with key problem areas including the following:

- Lack of staffing (nursing/consultant) and areas of limited oncology ‘engagement’
- Lack of ‘fast track’ options to avoid A & E
- Limited induction training and education (and engagement) for acute/emergency care physicians & nurses
- Poor information systems, limited flagging systems
- Poorly developed emergency cancer pathways and audit cycles (MSCC, FN)

Solutions and options for acute oncology in England require effective leadership and a clear understanding of cancer patient pathways within cancer networks but also within individual hospital trusts. The different models that will need to be developed depending on local service configurations may be very different nationally but all will share the common themes of triage, cancer alerts, early specialist review and defined inpatient pathways; areas that have all been highlighted by the NHS improvement, transforming inpatient care programme [5].

The ACP and medical oncology (MO) have a wide role and responsibility for AO development across many aspects of cancer care.

**New Cancers:** AO teams have a key role in planning the services for all cancer patients who present via the emergency route and have a potentially powerful role to ensure that urgent cancer referrals which do not fit existing routes, are seen promptly in the appropriate services. We should consider developing novel fast track services with key partners (urgent fast track capacity, working alongside acute medical units, etc).

**Treatment complications:** This is core AO function. In units with non-resident oncology, we need greater MO resource to improve emergency pathways, service development and audit but built around acute care hubs. In Units with resident oncology, we need to consider additional training for MOs who work in cancer admission units (Centres) facing acute medicine presentations.

**Cancer complications:** This group represent the largest proportion of emergency patients and often present with complex issues including comorbidity, progressive cancer and end of life care (EOL) needs. AOS have a leading role in the assessment of specific complications as outlined in national peer review. For many patients, the AOT should outline the care pathway and discharge to appropriate services (e.g. palliative care, cancer site-specific teams, primary care) to ensure optimal efficiency and effectiveness of the service.

**Education and research:** MO has an important role in developing and providing emergency cancer medicine education – ACP leadership on protocols, AO training modules, publications, etc. Adults with cancer should be offered and treated on a clinical trial where a clinical trial for their particular cancer is available. This principle holds true for AOS who should support clinical trials on all aspects of emergency cancer medicine. MO is well placed to lead on AO research.

### Admission avoidance

Future strategies for affordable and quality care are increasingly focussed on admission avoidance and seamless care extending into the community (Future Hospital Commission, Emergency Medicine Strategy, NHS England). MO needs to drive service innovation in this area including comprehensive triage, access to ambulatory care, and community AO initiatives. Patients with a known cancer diagnosis and health professional should have access to urgent telephone advice with an option to sign post for appropriate emergency care or offer admission avoidance strategies including patient self-management advice, community review (home/GP, pall care/DN), access to ambulatory care or day case facility (within 24 h) or urgent OPD review (within 7 days). Triage by appropriate professionals, including specialist nurses, should be strengthened by senior MO input. AOS should align with acute medicine in the development of acute care hubs, admission avoidance strategies, and service development – MO needs to work closer with acute medicine ambulatory care units to facilitate joint working. MO leadership in AO is well placed to influence cancer strategy and support community based initiatives that promote early referral and contingency planning in AO [6].

### Actions

- Complete an acute oncology service specification in 2015 and work to deliver a nationwide Acute Oncology service.
- Continuing the development of training and CPD in acute oncology (published text 2014).
- Develop the national network of expertise in acute oncology.

### Measures

- Consultant numbers and named AO leads.
- The development of peer review measures for AO.
- Development of demonstrator sites for ambulatory care & urgent cancer referral.
- Development of an acute oncology forum and workshops.
- Service outcome measures:
  1. Number of emergency admissions for cancer and types (I–III)
  2. Inpatient length of stay for all non-surgical emergency cancer patients
  3. Mortality in neutropenic sepsis according to risk stratification group
  4. Time to first antibiotic in neutropenic sepsis: ‘door to needle time’
  5. Deaths within 30 days of receiving chemotherapy and radiotherapy
  6. Annual patient and professional experience survey for all emergency patients
  7. Time from emergency admission to cancer diagnosis and to clear management plan

## Figures and Tables

**Table:** 

Key requirements of an acute oncology service
A service that brings together the multiprofessional team with the skills and expertise to care for acutely ill cancer patients. All hospitals that receive emergency admissions should have access to an AOS, 24 hours per day and 7 days per week. Establishment of excellent working relationships between the acute oncology team, emergency services, and acute medical and surgical specialties and palliative care teams. Appropriate levels of Consultant Oncologist cover (either clinical or medical oncology) to ensure AOS peer-review compliance and senior decision-making 24/7. Appropriate levels of specialist acute oncology nurse time to support 7-day services. Adequate timely access to acute diagnostic services including acute imaging capacity. Single point of contact to the acute oncology team for specialist teams, such as a single phone number and/or a defined clinical location. 24/7 access to telephone advice from trained staff. Management protocols for oncological emergencies available to A & E and all acute specialties. These should, as far as possible, be consistent nationally. Regular audit of activity and key outcome measures. Excellent acute oncology educational training. Fast-track access to specialist oncological clinics from A & E and primary care to avoid inappropriate emergency admissions. Flexible models of ambulatory care to drive admission avoidance and seamless care across the hospital/community interface.

## References

[1] Mort D, Lansdown M (2008). For better or worse? A review of the care of patients who died within 30 days of receiving systemic anti-cancer therapy.

[2] (2009). National chemotherapy advisory group (NCAG) “Chemotherapy services in England: ensuring quality and safety”.

[3] Marshall E, Young A, Clark P, Selby P (2013).

[4] Young A, Marshall E, Robinson B Responding to acute care needs of cancer patients: recent trends across continents.

[5] www.nationalpeerreview.nhs.uk.

[6] King J, Inghan–Clark C, Parker C (2011). Towards saving a million bed days: reducing length of stay through an acute oncology model of care for inpatients diagnosed as having cancer. BMJ Qual Saf.

# Brief report

**Published:** 2016

## 8. The care of older cancer patients

**Dr Alistair Ring, Dr Janine Mansi, Dr Danielle Harari, Dr Tania Kalsi, and Professor Peter Selby**

Almost all medical oncologists will continue to be involved in the care of older patients with cancer. We will work to develop improved care for older cancer patients closely with geriatricians and all other healthcare disciplines and professions and encourage more medical oncologists to develop special interests in the development of this area.

### Background

In United Kingdom and most developed countries, the population is ageing. The proportion of people in the United Kingdom aged 65 and over increased from 15% in 1985 to 17% in 2010, representing an absolute increase of 1.7 million people. By 2035, it is projected that those aged 65 and over will account for 23% of the total UK population and that there will be 3.5 million people aged 85 or over living in the UK [1].

At present, 164,000 people aged 70 and over are diagnosed with cancer every year in the United Kingdom, representing 50% of all cancer diagnoses [2]. With the ageing of the population both the proportion of cancers diagnosed in older patients and the absolute number of cancer diagnoses in this patient group is likely to rise, such that by 2030 it is anticipated that 70% of cancers will occur in people aged over 65 [3].

There is increasing discussion about the service needs and developments for cancer care for older people [4–7]. The International Cancer Benchmarking Partnership (ICBP) and EUROCARE studies suggest that the survival gap is widening between older and younger patients in Europe and there are worrying indications within these studies that UK older patients may be relatively disadvantaged [8–10].

### Diagnosis and treatment

It has been proposed that one reason for the apparent poor survival of older patients with cancer is delays to diagnosis and late stage at presentation [11, 12]. A number of initiatives have been undertaken or are underway to improve symptom awareness, self-referral and presentation to screening and diagnostic services [13, 14]. Such interventions are a vital component of any strategy that aims to improve the outlook of older patients with cancer.

Surgery remains the most important curative modality for cancer patients and is appropriate for many older patients. In a 2011 report from the NCIN, there was a large reduction with age in the percentage of patients receiving a major resection for cancer [15]. The reductions were apparent even for patients aged over 50 years, but for patients aged 80 and over, less than 2% had a record of a major resection for six of the 13 cancer sites analysed. The evidence to support decision-making in this age group remains limited but Korc-Grodzicki and others have emphasised that ‘chronological age alone should not be a determinant for treatment decisions’ [16]. Older patients can do very well and careful selection based on functional assessment, comorbidity, frailty and the evaluation of perioperative risks is essential.

Despite the importance of appropriate radiation treatment in all patients with cancer, including older patients, the uptake of this treatment may be relatively low [17, 18]. When considering the role of radiotherapy, it is important to take into account the relatively low risk of locoregional recurrence in some tumour types in older patients, particularly if the risks of death from competing causes is high [18]. Where radiotherapy is deemed appropriate consideration needs to be given to the pre-emptive management of toxicities, and the use of hypofactionated regimens which may minimise inconvenience if travel to the radiotherapy treatment centre is difficult.

Systemic therapy in the forms of cytotoxic chemotherapy and biological agents has a role to play in most tumour types. Contemporary therapies, with judicious use of supportive treatments, such as growth factor support, have the potential to be much less toxic than historical regimens, and hence may be better tolerated in older patients. Despite this, the evidence suggests that older patients are under-treated [19]. This lack of equity in access and uptake of cancer care may be leading to poorer outcomes. For examples, in prostate cancer, greater than 70% of cancer deaths occur in men aged over 75, usually with a more aggressive disease, yet few older patients receive treatment for localised prostate cancer and are, in the majority of cases, denied access to chemotherapy for advanced disease which if carefully selected can confer benefits with avoidable toxicity [20, 21]. Colorectal cancer is another disease of the older adult, yet the evidence again suggests that optimal therapy is not always being provided to this patient cohort [22–24]. A significant proportion of older women with triple negative breast cancer receive less chemotherapy than their younger counterparts and older women may even receive less endocrine therapy than their younger counterparts with breast cancer [25–27].

In United Kingdom, a National Cancer Equality Initiative (NCEI)/Pharmaceutical Oncology Initiative (POI) joint report concluded that ‘clinicians may over rely on chronological age as a proxy for other factors, which are often but not necessarily associated with age, for example co-morbidities and frailty’ [28]. The recent NHS England publication ‘Are older people receiving cancer drugs?’ demonstrates considerable variation in the use of systemic anticancer therapy (SACT) in older people and concludes: ‘It does not seem plausible that differences in referral patterns or the age profile of populations served by hospitals could alone explain the variation. The reason for this variation requires further exploration’. It seems likely that some variation at least will be caused by the use of age as a proxy for clinical factors, rather than differences in patient health status or preference’ [19]. There may be good clinical reasons why some older patients receive less intensive treatment: they are more likely to have comorbidities and some may be less able to tolerate the side effects of surgery, radiotherapy, and chemotherapy. Furthermore, some older patients may choose not to pursue active treatment. However, chronological age alone may be a poor proxy for treatment tolerance; many older patients have little in the way of other health problems and may tolerate treatment just as well as their younger counterparts. Treatment discussions are better informed based on measures of ‘fitness’ or ‘biological’ age and not chronological age in isolation.

Biological age is likely to be best determined by some form of Comprehensive Geriatric Assessment or ‘CGA’. This might include measurements of comorbidities, functional status, cognition, nutrition, psychological state, and social support [29]. Such measures may have a useful role to play in predicting tolerance of treatment and adverse outcomes, as an aid to shared decision-making [29–31]. Whilst there are a number of scales available to measure each of these domains, there is little consensus on which domains to include and which measurement tools to use. For instance, we have no accepted tools to identify frailty yet. A consensus as to the most appropriate measures will be needed before progress can be made, and this must recognise that at present, there are very few tools that have been robustly validated against clinical outcomes. As such, we should be cautious about applying them outside of a research setting.

### Research

Developments to optimise evidence-based treatment will inevitably need to be underpinned by clinical trial evidence. Recognising the disparities that exist for cancer care in older people, the European Organisation for Research and Treatment of Cancer (EORTC) established an EORTC Cancer in the elderly task force (ETF), with the stated aim of improving access to clinical trials and research in order to deliver optimum standards of care for the geriatric population. A joint position paper between the EORTC, the alliance for clinical trials in oncology and the international society of geriatric oncology specifies a roadmap for research and clinical trials in older people and emphasising the absolute requirement for clinical trials to be without an upper age limit, thus removing a critical barrier for the eligible older patient [32, 33]. They also recommend the need for standardised approaches to the measurement of frailty and comorbidity in trials and practice [32]. A further focus of research interest will be the definition of outcome measures, in particular with respect to toxicity. While grade 3 and 4 toxicities are routinely reported in clinical trials, lower grade toxicities may determine treatment modifications/discontinuation, particularly in older patients [34]. To date, in the absence of evidence from RCTs, evidence based medicine has not become routine or possible for many older patients with cancer. In some settings, alternatives to RCTs may become essential. Indeed prospective cohort studies with relatively permissive inclusion criteria may have the advantage of minimising selection bias associated with some RCTs. An additional strand of research will be whether biological markers of ageing, such as telomere length and markers of chronic inflammation, will improve our understanding of the biology underlying cancer in older patients and add to the information provided by clinical assessments of biological age [35]. Future research to improve outcomes for older patients with cancer will be highly multidisciplinary and include oncologists of all specialties, geriatricians in medicine and allied professions, methodologists, statisticians and biomedical scientists.

### Education and training

A recent survey of medical oncologists in training identified that many (66%) had never received any specific training on particular needs of elderly patients with cancer, and that only 27% felt confident in assessing risk to make treatment decisions in older patients compared with 81% being confident with treatment decisions in younger patients [36]. A change in the medical oncology curriculum has been made to reflect this, and we recognise that further changes may become necessary as the field evolves.

### The multidisciplinary team

We need multiprofessional approaches to care for older patients with cancer that consider patient choice and evaluate frailty and not age alone. There is a need for the development and application of geriatric decision making tools and their recognition and routine use in oncology. This will require the expertise of the geriatric multidisciplinary team. This extends to include physiotherapists, occupational therapists, dieticians, social workers, as well as care of the elderly physicians. It is also important to recognise that a formal geriatric assessment should not be seen simply as a signal to either initiate a therapy or not, but as a pathway to identify reversible health problems which, if addressed, might mean that the patient becomes fit enough for treatment. Therefore, if such assessments are to be conducted, there should be established pathways in place for onward referral to specialist multidisciplinary services. Moreover, these pathways need to be accessible and interventions implemented in a timely manner in order that subsequent anticancer therapy can be delivered in an acceptable timeframe. Such improved general patient care and better tailoring of treatment may require upfront expenditure but yield improved survival and quality of life, reduce treatment complications and resource use, reduce dependency, and carer burdens. To achieve all, this will need integration of healthcare and social care for older patients with cancer.

### Moving forward: the agenda in the United Kingdom

In United Kingdom, the publication of the NCEI-POI joint report highlighted above [28] has been part of a concerted recent effort to redress the balance in favour of the older cancer patient, culminating in the launch of an ‘Action for the Elderly in Cancer’ initiative as the main priority of the NCEI at the Britain Against Cancer Conference in London (2014). Although geriatric oncology is beginning to become established as a specialty in North America and Europe this specialist approach is not yet widely available in United Kingdom. The approaches that are developed towards managing cancer in older people will have a profound impact on future cancer policy and outcomes. While cancer survivorship is increasing overall, with the most recent figures indicating 11.7 million cancer survivors in the United States and nearly 14 million in Europe, the percentage is lower in older people compared with the overall population. Thus, cancer survivorship may plateau or even decline, unless we develop better approaches for the management of older patients with cancer.

### Actions

The ACP will undertake a series of initiatives to address the needs of older patients with cancer:

- Publication of the ACP ‘Problem Solving in Older Cancer Patients’ book in 2016 [37].
- Lobby for a national initiative to develop innovative services for older patients with cancer and develop new roles for oncologists who will take a specific interest and support and advise their colleagues in their work.
- **Research and innovation.** We will emphasise the importance of research and innovation to improve the evidence base upon which we work. This may involve RCTs to include older patients but pragmatic non-randomised studies and modelling studies may be needed. We recognise that any research involving older patients with cancer should be conducted in collaboration with elderly care physicians.
- **Education and training.** We will continue to develop further our programme of CPD in this area. We hosted (with a sponsorship from Macmillan Cancer Support and Cancer Research UK) in October 2014, a workshop on ‘Cancer Care in Elderly Patients’. Working with Clinical Publishing and drawing on the proceedings of the workshop, we will sponsor and deliver a text to be called ‘Problem Solving for Elderly Cancer Patients’ to be published in 2015/2016 to promote continued learning in the area. We will continue to work with the geriatric community as well as our clinical oncology and palliative care colleagues in this area.
- **Collaboration.** Establishment of National and local collaborations with Care of the Elderly services, building upon our existing collaboration with the British Geriatrics Society.

### Measures

- Patient feedback surveys.
- Uptake of SACT in older cancer patients with changes over time and shared data between institutions.
- The number of recognised development teams for cancer care in older people with leadership teams with appropriate protected time for the work.
- Metrics for recruitment into clinical trials of older patients.

## References

[1] (2012). Population ageing in the United Kingdom, its constituent countries and the European union office for national statistics.

[2] Cancer research UK [Internet] Cancer incidence by age – UK statistics. http://info.cancerresearchuk.org/cancerstats/incidence/age/.

[3] Mistry M, Parkin D, Ahmad A (2011). Cancer incidence in the United Kingdom: projections to the year 2030. Br J Cancer.

[4] Lawler M, Selby P, Aapro M (2014). Ageism in cancer care; we need to change our mindset. BMJ.

[5] Lichtman SM, Hurria A, Jacobsen PN (2014). Geriatric oncology: an overview. J Clin Oncol.

[6] (2013). Global AgeWatch Index 2013 Insight report. http://www.helpage.org/global-agewatch/.

[7] Levit L, Balogh E, Nass S (2013). http://www.nap.edu/catalog.php?record_id=18359.

[8] Coleman MP, Forman D, Bryant H (2011). Cancer survival in Australia, Canada, Denmark, Norway, Sweden, and the UK, 1995–2007 (the International Cancer Benchmarking Partnership): an analysis of population-based cancer registry data. Lancet.

[9] Quaglia A, Tavilla A, Shack L, EUROCARE Working Group (2009). The cancer survival gap between elderly and middle-aged patients in Europe is widening. Eur J Cancer.

[10] De Angelis R, Sant M, Coleman MP, EUROCARE-5 Working Group (2014). Cancer survival in Europe 1999–2007 by country and age: results of EUROCARE—5-a population-based study. Lancet Oncol.

[11] Tate AR, Nicholson A, Cassell JA (2010). Are GPs under-investigating older patients presenting with symptoms of ovarian cancer? An observational study using general practice research database. Br J Cancer.

[12] Lyratzopoulos G, Abel GA, Barbiere JM (2012). Variation in advanced stage at diagnosis of lung and female breast cancer in an English region 2006–2009. Br J Cancer.

[13] Linsell L, Forbes LJ, Kapari M (2009). A randomised controlled trial of an intervention to promote early presentation of breast cancer in older women: effect on breast cancer awareness. Br J Cancer.

[14] Cancer Research UK. www.cancerresearchuk.org/health-professional/early-diagnosis-activities/national-awareness-and-early-diagnosis-initiative-naedi.

[15] (2011). Major surgical resections, England, 2004–2006.

[16] Korc-Grodzicki B, Downey RJ, Shahrokni A (2014). Surgical considerations in older adults with cancer. J Clin Oncol.

[17] Guadagnolo BA, Liao KP, Elting L (2013). Use of radiation therapy in the last 30 days of life among a large population-based cohort of elderly patients in the United States. J Clin Oncol.

[18] Smith GL, Smith BD (2014). Radiation treatment in older patients: a framework for clinical decision making. J Clin Oncol.

[19] NHS England (2013). Are older people receiving cancer drugs? An analysis of patterns in cancer drug delivery according to the age of patient. http://www.england.nhs.uk/wp-content/uploads/2013/12/old-people-rec-cancer-drugs.pdf.

[20] Aapro MS (2012). Management of advanced prostate cancer in senior adults: the new landscape. Oncologist.

[21] Fung C, Dale W, Mohile SG (2014). Prostate cancer in the elderly patient. J Clin Oncol.

[22] Luo R, Giordano SH, Freeman JL (2006). Referral to medical oncology: a crucial step in the treatment of older patients with stage III colon cancer. Oncologist.

[23] Hubbard J, Jatoi A (2011). Adjuvant chemotherapy in colon cancer: ageism or appropriate care?. J Clin Oncol.

[24] McCleary NJ, Dotan E, Browner I (2014). Refining the chemotherapy approach for older patients with colon cancer. J Clin Oncol.

[25] Aapro M, Wildiers H (2012). Triple-negative breast cancer in the older population. Ann Oncol.

[26] Tew WP, Muss HB, Kimmick GG (2014). Breast and ovarian cancer in the older woman. J Clin Oncol.

[27] Hershman DL, Shao T, Kushi LH (2011). Early discontinuation and non-adherence to adjuvant hormonal therapy are associated with increased mortality in women with breast cancer. Breast Cancer Res Treat.

[28] Department of Health (2012). The impact of patient age on decision making in oncology.

[29] Kenis C, Decoster L, Van Puyvelde K (2014). Performance of two geriatric screening tools in older patients with cancer. J Clin Oncol.

[30] Hurria A, Togawa K, Mohile S (2011). Predicting chemotherapy toxicity in older adults with cancer: a prospective multicenter study. J Clin Oncol.

[31] Revenig LM, Canter DJ, Taylor MD (2013). Too frail for surgery? Initial results of a large multidisciplinary prospective study examining preoperative variables predictive of poor surgical outcomes. J Am Coll Surg.

[32] Wildiers H, Mauer M, Pallis A (2013). End points and trial design in geriatric oncology research: a joint European organisation for research and treatment of cancer Alliance for clinical trials in oncology - international society of geriatric oncology position article. J Clin Oncol.

[33] Hurria A, Dale W, Mooney M (2014). Designing therapeutic clinical trials for older and frail adults with cancer: U13 conference recommendations. J Clin Oncol.

[34] Kalsi T, Babic-Illman G, Fields P (2014). The impact of low-grade toxicity in older people with cancer undergoing chemotherapy. Br J Cancer.

[35] Pallis AG, Hatse S, Brouwers B (2014). Evaluating the physiological reserves of older patients with cancer: the value of biological markers of aging. J Geriatr Oncol.

[36] Kalsi T, Payne S, Brodie H (2013). Are the UK oncology trainees adequately informed about the needs of older people with cancer?. Br J Cancer.

[37] Ring A, Harari D, Kalsi T, Mansi T, Selby P (2016). Problem solving in older cancer patients.

# Brief report

**Published:** 2016

## 9. New ways to ensure the most timely access to best care

**Professor Richard Neal, Professor Peter Selby, and Professor Johnathan Joffe**

Support new ways of working and the reconfiguration of services in order to provide the best and most timely access to investigations, prompt diagnosis and prompt treatment by the appropriate specialised team for all patients.

Despite encouraging progress, UK outcomes lag behind some international comparisons. Evidence suggests that this is, at least in part, because of delays in diagnosis which reflect delays in presentation, investigation, and referral [1a, 1b].

A more comprehensive integration between primary care and other parts of cancer care is needed to improve the timeliness of access to diagnosis and treatment for patients with cancer and to ensure we make appropriate responses to patients with symptoms which might indicate cancer. There is actively ongoing work on this topic by the International Cancer Benchmarking Partnership and the National Institute for Health and Care Excellence, which is due to publish its updated cancer referral guidelines in June 2015.

There is evidence that early diagnosis not only improves clinical outcomes [2] but may reduce overall service costs [3]. A combination of low awareness of potential cancer symptoms and more negative beliefs about cancer in the UK populations, compared with other similar countries, are likely to contribute to delayed presentation with cancer symptoms, leading to advanced stage at presentation and a smaller chance of survival [4]. There is also emerging evidence from the English ‘Be Clear On Cancer’ campaign that a nationally coordinated, multicomponent, awareness campaign (at least for lung cancer) is likely to positively influence outcomes across the whole English National Awareness and Early Diagnosis Initiative (NAEDI) pathway [5].

Traditional models of cancer care depend on referral from primary care to local secondary care with referral to specialist tertiary care as and when appropriate. Within these models, the integration of specialist support from other hospital based teams to support efficient patient diagnosis and the effective management of co-morbidity is often poorly defined. Although medical oncologists are usually not doctors of first contact for patients with symptoms which may indicate cancer, ACP can promote the development of ‘integrated’ cancer services to strengthen both ‘vertical’ integration between primary, secondary, tertiary and social care services for patients with cancer and ‘horizontal’ integration between cancer services and other hospital specialties which are critical to excellent cancer care including diagnostic services, across all medical specialities. It is well recognised that many referrals initially go to the ‘wrong specialty’, hence integration is essential. We believe this is timely not only because there is large and pressing clinical need for service developments which are excellent and financially sustainable, but also because the development of new technologies in health informatics and telemedicine and the monitoring and provision of healthcare for cancer patients and for their follow up, have moved to a point where they may be deployed effectively within healthcare models.

A key component of our discussions should be the application of modern health informatics to provide the information flow which ensures excellence in cancer care; improved patient knowledge and empowerment; timeliness of access to care and cost-effective and financially sustainable monitoring and follow-up services; explores new informatics and computing technologies to support the appropriate collection of data as close as possible to the patients’ home; the application and novel technologies for tele-oncology; minimising hospital stay by maximising the quality and safety of follow up that can be provided in the community supported by data collection and data transfer.

The English National Awareness and Early Diagnosis Initiative (NAEDI) was set up in 2008, following the publication of the Cancer Reform Strategy [6]. It aimed to understand and address the reasons for late diagnosis of cancer in England [7]. This has focused on delays in primary care, delays post-referral from primary care, and cancer symptom awareness. Its work covers various domains including awareness raising, a primary care audit of diagnoses, supporting primary care, system change, and innovations and evaluative and research.

The impact of this on improving cancer diagnosis in general practice has recently been summarised [8]. This concluded that specific primary care initiatives promoted by cancer networks, had ‘an additional and positive impact on urgent referrals for suspected cancer’. These initiatives included participation in quality improvement activities including clinical audit, significant event analysis, use of risk assessment tools, and development of practice plans. There has been a huge increase in the amount of UK early diagnosis research in recent years – this is adding to our understanding of reasons for late diagnosis and interventions to overcome them [9].

More recent activity, through the English Department of Health, has focused on the Accelerate Coordinate and Evaluate programme. This aims to build on service and pathway development activity in England in order to improve early diagnosis and, through robust evaluation, inform the commissioning intentions of the future. Initial pilots have been commissioned and will be evaluated.

Colleagues in Denmark have identified relatively poor cancer outcomes and possible delays in diagnosis and over many years set out efforts to tackle the perceived delays [10]. A brief survey of their work indicates careful studies that have addressed the issues of diagnostic delay in specific cancers such as gynaecological malignancies and colorectal cancer [11, 12]. Danish investigators have studied generic issues underlying delay in diagnosis such as the potential for adverse consequences from gatekeeper practices [13], the prevalence of cancer alarm symptoms [14], the impact on patients’ confidence in their GP [15], international comparisons in the international benchmarking programmes and comparisons with the United Kingdom [16]. Their work has underpinned consensus statements such as the Aarhus statement on improving design and reporting studies on early cancer diagnosis [17]. Useful commentaries, often in collaboration with UK-based authors, have emerged from this work [18, 19].

In 2007 following the recognition of the lack of progress in the previous decade, the government and Danish regions launched a new diagnostic strategy, the key components of which were as follows:

- Cancer should be dealt with as an acute condition. If the GP or another doctor suspects cancer, only medically necessary waiting times should be accepted in the clinical pathway from symptom to treatment.
- Danish Regions established the service target that a patient should be seen within 2 days following a GP referral with suspicion of cancer.
- Multidisciplinary working groups, chaired by the National Board of Health, were established to describe the ideal clinical pathway for each of the common cancer types. These included maximum acceptable waiting times at each phase of the pathway beginning from the time of referral.
- The government gave the National Board of Health the task of measuring and reporting waiting times.
- A commitment was made to reduce bottlenecks in GP access to diagnostic investigations and to help GPs in difficult cases.
- A commitment was made to invest in necessary equipment.

Examples of potential benefits may include reduction in the proportion of cancers diagnosed at late stage; improved care of co-morbidity and frailty in cancer patients; improved monitoring of patients on treatment; and improved follow-up and long-term monitoring. Initial data on diagnostic intervals has shown that patients referred via the new cancer patient pathways were diagnosed quicker, although those who were diagnosed through other routes were diagnosed more slowly [20].

Recommendations in this area are expected from the UK Taskforce on Cancer Services.

### Actions

- ACP workshop on patient-centred and integrated cancer care in October 2016.
- Support for the recommendations expected in the taskforce report in 2015, support individual local initiatives and sharing proposals nationally.

### Measures

The measures ideally using nationally agreed metrics may include the following:

- Reduced emergency and late stage diagnoses.
- Shifts in stage at diagnosis towards a greater proportion of stage I and stage II cases.

## Figures and Tables

**Figure 1.** Data from the CONCORD-2 study shows evidence of improving one year and five year survival for patients from six jurisdictions. Comparison of the UK outcomes for colon cancer, lung cancer and breast cancer show that, despite continued improvement in the UK, there is still a lag behind the results of the five comparator jurisdictions. Data provided and prepared by Professor Michel Coleman and the CONCORD-2 team. [Allemani C, Weir HK and Carreira H *et al* (2015) Global surveillance of cancer survival 1995-2009: analysis of individual data for 25,676,887 patients from 279 population-based registries in 67 countries (CONCORD-2) *Lancet* 385(9972) 977–1010 DOI: 10.1016/S0140-6736(14)62038-9]

## References

[1a] Coleman MP, Forman D, Bryant H (2011). Cancer survival in Australia, Canada, Denmark, Norway, Sweden, and the UK, 1995–2007 (the International Cancer Benchmarking Partnership): an analysis of population-based cancer registry data. Lancet.

[1b] Allemani C, Weir HK, Carreira H (2015). Global surveillance of cancer survival 1995–2009: analysis of individual data for 25,676,887 patients from 279 population-based registries in 67 countries (CONCORD-2). Lancet.

[2] Neal RD, Tharmanathan P, France B (2015). Is increased time to diagnosis and treatment in symptomatic cancer associated with poorer outcomes? Systematic review. Br J Cancer.

[3] An analysis of the financial implications of achieving earlier diagnosis of colorectal, lung and ovarian cancer.

[4] Forbes LJ, Simon AE, Warburton F (2013). Differences in cancer awareness and beliefs between Australia, Canada, Denmark, Norway, Sweden and the UK (the International Cancer Benchmarking Partnership): do they contribute to differences in cancer survival?. Br J Cancer.

[5] Ironmonger L, Ohuma E, Ormiston-Smith N (2014). An evaluation of the impact of large-scale interventions to raise public awareness of a lung cancer symptom. Brit J Cancer.

[6] (2007). Cancer reform strategy.

[7] Richards MA (2009). The national awareness and early diagnosis initiative in England: assembling the evidence. Br J Cancer.

[8] Rubin G, Walter F, Emery J (2014). Research into practice: earlier diagnosis of symptomatic cancer. BJGP.

[9] Hiom SC (2015). Diagnosing cancer earlier: reviewing the evidence for improving cancer survival. British Journal of Cancer.

[10] Hansen RP, Vedsted P, Sokolowski I (2011). Time intervals from first symptom to treatment of cancer: a cohort study of 2,212 newly diagnosed cancer patients. BMC Health Serv Res.

[11] Vandborg MP, Christensen RD, Kragstrup J (2011). Reasons for diagnostic delay in gynaecological malignancies. Int J Gynecol Cancer.

[12] Tørring ML, Frydenberg M, Hansen RP (2011). Time to diagnosis and mortality in colorectal cancer: a cohort study in primary care. Br J Cancer.

[13] Vedsted P, Olesen F (2011). Are the serious problems in cancer survival partly rooted in gatekeeper principles? An ecologic study. Br J Gen Pract.

[14] Svendsen RP, Støvring H, Hansen BL (2010). Prevalence of cancer alarm symptoms: a population-based cross-sectional study. Scand J Prim Health Care.

[15] Larsen MB, Hansen RP, Olesen F (2011). Patients’ confidence in their GP before and after being diagnosed with cancer. Br J Gen Pract.

[16] Maringe C, Walters S, Rachet B (2013). Stage at diagnosis and colorectal cancer survival in six high-income countries: a population-based study of patients diagnosed during 2000–2007. Acta Oncol.

[17] Weller D, Vedsted P, Rubin G (2012). The Aarhus statement: improving design and reporting of studies on early cancer diagnosis. Br J Cancer.

[18] Rubin G, Vedsted P, Emery J (2011). Improving cancer outcomes: better access to diagnostics in primary care could be critical. Br J Gen Pract.

[19] Hamilton W, Vedsted P (2011). Cancer and primary care: the clinical and research agenda. Br J Gen Pract.

[20] Jensen H, Tørring ML, Olesen F (2015). Diagnostic intervals before and after implementation of cancer patient pathways – a GP survey and registry based comparison of three cohorts of cancer patients. BMC Cancer.

# Brief report

**Published:** 2016

## 10. Informatics and the delivery of new models of care

**Dr Geoff Hall, Dr Richard Griffiths, and Dr Adam Dangoor**

Work to improve care through the development of appropriate state-of the art informatics.

Accurate data on cancer diagnosis, treatment, toxicity, and outcomes are absolutely essential for improving cancer care and outcomes. The integration of large datasets under rigorous governance and regulation is a key tool to help us understand the needs of each patient and identify ways to improve services.

### Electronic systems, electronic records

The delivery of high-quality care to cancer patients requires the integration of a wide range of clinical information from a variety of disparate sources. Within hospitals, this almost always includes a multiplicity of trust-based systems each designed to deliver a specific element of the case notes (radiology, pathology, blood results, patient demographics, chemotherapy). The nature of most cancer patient journeys also demands the integration of information from a range of healthcare providers from primary care, community based nursing teams to cancer units, and regional cancer centres. Traditionally, this information, despite being created by electronic systems, has been collated through the creation of paper-based records combined into a traditional paper-based case notes.

The ACP believes that this model is no longer fit for purpose and will where possible work with both commissioners and deliverers of cancer services to ensure the use of integrated electronic patient records shared between providers. This will facilitate the integration of clinical decision support and error checking, allow referrals and orders to occur in real time, enhance patient access and engagement, facilitate audit and research and has the potential to improve compliance with national data collection such as Cancer Outcomes Services Dataset (COSD) and the Systemic Anti-Cancer Therapy dataset (SACT) [1, 2]. The first element of this strategy is where possible to ensure that all relevant cancer information regarding diagnosis, treatment and follow-up is created through the use of electronic systems rather than hand-written paper-based systems. To this end, the ACP will propose the mandated use of electronic prescribing systems for systemic anti-cancer therapy by April 2017. More ambitiously, the ACP would encourage medical oncologists to advocate the adoption of electronic health records for clinical noting, referrals and the communication of orders and results by April 2018.

### Shared data, shared systems

With the universal use of NHS number, all relevant data can and should be linked to allow clinicians access to all available data through a single common portal or ideally a single electronic health record (EHR). The cancer record should however not be developed as a stand-alone system, distinct from the patients other health records. The ACP believe it is essential that data relevant to patients’ cancer diagnosis is integrated into a hospital-wide EHR that links readily to primary care databases to ensure that effective communication between specialties/services within any individual healthcare provider is not compromised. Although no individual system or supplier is supported or recommended by the ACP, the systems must be capable of sharing data using internationally agreed standards or cloud-based data repositories to facilitate the exchange of information between organisations. The ACP encourage an open approach in developing these systems encouraging the sharing and adoption of best practice including the use of open-source software where this delivers a best-of-breed solution.

The ACP will work to ensure the implementation of electronic transfer of documents and data between organisations to avoid the delays which result from the use of post and traditional paper records. Ideally, the ACP will work with the Academy of Royal Colleges to promote the development of a single comprehensive health record which crosses organisational boundaries and ensures all members of the patient’s healthcare team have access to all the relevant data. Data exchange between hospital-based systems and primary care must be two-way. In addition to ensuring the community team have access to relevant data about the cancer and its treatment, it is imperative the hospital have access to relevant comorbidity, that is, the context within which the cancer is treated. Software design will need to ensure that while systems are able to collect the required complexity of data for specialist care, they can also generate appropriate summaries relevant for others. Software tools also need to be developed to help summarise and facilitate the interpretation of complex records. An example of this is the creation of an e-frailty index from primary care records, the transfer of which may guide the delivery of appropriate health-care to individuals with complex comorbidity, in particular elderly patients with cancer.

### Patient access, patient involvement

The ultimate extension of sharing data will be to define a mechanism which allows patients to securely access their health record. Towards this end, the ACP will mandate the adoption of Royal College recommendations to ensure patients are offered copies outpatient consultations, treatment summaries, multi-disciplinary team plans and hospital discharge summaries. The ACP proposes that this is seen as a key marker of quality care by commissioners and external reviewers of services. Ultimately, we would wish to see patients have secure access to their electronic health record summarising their diagnosis and treatment. The ACP supports the work of Cancer Research UK and local innovations such as the Birmingham patient portal and wish to see such systems become more widespread.

With secure access, the ACP will also wish to promote the ability of patients to add information directly to their electronic health record. The development of web-based tools, such as the Leeds Q-Tool system, will allow clinical teams to collect coded data directly from patients.

### Telehealth, telemonitoring

The ACP wish to see the continued development of informatics solutions which improve our ability to monitor patients on treatment and during follow-up without the need for the patient to leave home. We need to embrace the concept of moving the data not the patient. The use of video conferencing should be exploited to allow on-line consultations between patients and one or more members of their health-care team [3]. Videoconferencing facilities for consultations and MDT working need to be upgraded to consistent high standards.

The ACP will work with academic groups and commercial companies to support the ongoing development of software and technology to facilitate the remote monitoring of patients with cancer on treatment and conduct robust research to ensure they add value to clinical care.

Examples of this include technology to facilitate the home-monitoring of blood counts for patients on chemotherapy and software tools to allow patient-reported symptoms and outcome measures to be collected on pre-defined schedules and pathways to facilitate the delivery of remote follow-up of patients with cancer.

### Secure data, secure analysis

The ACP recognises the fundamental importance of data security and the need to ensure the protection of personal and private healthcare data. We will therefore work with and on behalf of patient representatives to ensure the benefits of shared data analysis are appreciated by patients. We will work with national bodies such as the National Cancer Intelligence Network and the National Cancer Registration Service to deliver the maximum intelligence from the data collected. Data must be collected as a consequence of clinical care (not as a separate ‘industry’ in its own right) and must be used routinely to monitor the clinical and cost-effectiveness of new treatments and new pathways of care. Data access and software tools must be developed to facilitate a comparison of clinical outcomes including for example deaths within 30-days of chemotherapy across different organisations to ensure the adoption of best-practice across United Kingdom. To support these disparate analyses, it is imperative that each organisation is required to collect data once in a form common to all regional and national requirements, ideally through the use of electronic health records. To this end, the ACP propose the adoption of a common data model based on the Cancer Services Outcomes Dataset (CSOD) to ensure that healthcare providers are able to readily export, combine and compare data between organisations without the need for complex data manipulation or transformation. All organisations wishing to use this data (e.g. COSD, cancer registration, national audits, SACT) must conform to this standard.

### Clinical systems, clinical engagement

There are a significant number of national drivers forcing the informatics agenda in healthcare leading to Trusts investing heavily in their IT infrastructure. Without strong clinical leadership, these projects are likely to fail to deliver on the intended benefits. In fact, one of the key reasons for the failure of the National Program for IT was poor clinical engagement. The ACP supports the national agenda for all Trusts to have a Chief Clinical Information Officer to provide clinical leadership for informatics projects [4]. The ACP urges medical oncologists to become involved in the procurement, design and deployment of informatics systems to ensure that the systems are fit for purpose for clinicians and cancer patients alike.

### Next-generation informatics

The last 20 years has seen massive advances in the molecular characterisation of cancer through technologies such as next-generation sequencing. The ACP believe that a similar focus needs to be applied to health informatics to maximise the benefit of new technology in this area. The ACP believes that the technology must adapt to the clinicians not an expectation for clinicians to adapt established ways of working. To this end, the analysis of plain-text with natural language processing and self-learning platforms, such as IBM’s Watson, must be assessed and implemented to facilitate and enhance the collection of a coded dataset collected through automatic rather than manual data extraction [5].

The ACP would also want to see medical oncology at the forefront of new technologies including the development and use of ‘wearable technology’, mobile heath ‘apps’, rapid learning systems [6], and advanced clinical decision support.

### Actions

- ACP workshop on health informatics in cancer care 2016
- Strengthen research, governance and leadership in Informatics in medical oncology

### Deliverables and monitoring

1. The ACP will seek wide support for a common dataset for all national data collection on cancer in 2016. In 2017, an agreed data model for cancer will be developed and published.
2. The ACP will work to ensure that patients have access to their medical records, by 2016. By 2017, the ACP wishes to see the wide-spread introduction of electronic access to electronic health records.
3. Work with commissioners and suppliers of cancer services to ensure the exclusive and nationwide use of electronic prescribing systems to support the delivery of chemotherapy.
4. Work with the NCIN to see the development of a portal of data driven presentations of clinical outcomes which can be configured at national, regional and local level.

## References

[1] http://www.ncin.org.uk/collecting_and_using_data/data_collection/cosd.

[2] http://www.chemodataset.nhs.uk/home.

[3] http://digital.innovation.nhs.uk/dl/cv_content/32200.

[4] http://www.ehi.co.uk/campaign/ccio-home.cfm.

[5] http://www.ibm.com/smarterplanet/us/en/ibmwatson/watson-oncology.html.

[6] Abernethy AP1, Etheredge LM, Ganz PA (2010). Rapid-learning system for cancer care. J Clin Oncol.

# Brief report

**Published:** 2016

## 11. Cancer survivors

**Dr Jeff White, Professor Michael Hawkins, Dr Rod Skinner, Professor John Radford, Professor Adam Glaser, and Dr Dan Stark**

All medical oncologists will make contributions to the planning and delivery of services for cancer survivors and some will develop special interests in this area.

The definition of ‘cancer survivor’ varies, for example the National Cancer Institute [1] defines this as a patient with cancer from diagnosis to end of life while the National Cancer Survivor Initiative defined it as ‘anyone living with and beyond cancer’ (http://www.ncsi.org.uk/). However, most health professional utilise the term for the period post active treatment and thus The Institute of Medicine’s analysis of the Cancer Care Trajectory, with the focus on cancer-free survival and the management of chronic or intermittent cancer which has become an increasing part of cancer care (Figure 1) is a useful definition.

Recent data from Cancer Research UK [3] suggest that of those patients diagnosed in 2010/2011 over 50% will become long-term survivors. Even those patients not cured now experience increasing survival with advanced cancer. With an ageing population, there will be increased numbers of cancer survivors in United Kingdom, 2 million in 2010 rising to 4 million by 2030. There are increasing numbers of very long-term survivors of cancer. Moreover, the needs of these cancer survivors are further complicated by the age-associated increase in co morbidity.

The identification of the particular issues cancer survivors experience, which can be grouped as physical and psycho social, began in the 1980s with the National Coalition of Cancer Survivorship (NCCS) in the USA [5]. Since that time there has been increasing recognition of the issues surrounding survivorship; these are most developed in the long-term survivors of paediatric cancers, such as the frequency of late adverse effects of cancer treatment [4]. Much of the work required in this area can be based upon the principles of survivorship established in paediatric oncology, for example from the North American Childhood Cancer Survivor Study, which demonstrates that over 70% of such survivors suffer from at least one chronic health condition by 30 years from cancer diagnosis [4] and the importance of developing sustainable long-term follow-up strategies [6].

Increased survival after cancer does not necessarily equate with a good quality of life. In a prospective longitudinal study of un-met needs [7] in common cancers, 30% of patients had more than 5 un-met needs at end of treatment and 60% of these persisted 6 months later. Some of these needs will be obvious such, as physical symptoms and perhaps less readily appreciated psychological needs; other needs may not be recognised by health care staff, for example, the impact on employment and finances. Moreover, there is evidence to suggest cancer survivors experience employment related problems, which have detrimental effects on various QOL measures [8]. Such topics may be seen as the domains of multiple care disciplines; this along with the fact that these needs change with the trajectory of the cancer journey means service design and research in cancer survivorship are challenging, made even more complex by the need to integrate across multiple agencies.

Although psychological distress is common in cancer survivors, the evidence that interventions make a difference is variable [9, 10]. The variance may arise due to methodological issues and suggests an important need to improve the quality of research to optimise outcomes and applicability of such interventions.

Cochrane reviews of the evidence for interventions in survivorship is sparse, for example, in breast cancer the evidence for multidimensional programmes is relatively low- and for short-term gains; a recent expert report of a meta-analysis of the effect of weight, diet, and physical activity on incidence, recurrence, and mortality was unable to make strong recommendations about lifestyles issues [11], indicating the important need for further evaluation.

There is some evidence that these needs vary with the age of the patients, for biological and other reasons. There have been valuable analyses of the issues presented by cancer survivors, with an especial focus on childhood and teenage and young adult cancer patients who survive to adult life [12–15]. For long-term survivors of cancers that are commoner in adult life (breast, gynaecological, prostate, and colorectal cancers). Harrington *et al* [16] conducted a systematic review of the literature to describe the symptoms experienced by long-term cancer survivors and noted that depressive symptoms, pain, and fatigue were commonly found in cancer survivors. Bellury and colleagues [17] explored the issues for cancer survivorship in elderly patients with cancer and in their review noted that cancer survivorship among the elderly is quantitatively and qualitatively different from cancer survivorship among other age groups and created a conceptual model of elderly cancer survivorship. Brearley *et al* [18] identified four main gaps in our knowledge relating to the practical and physical problems associated with cancer survivorship including the identification of the key symptoms, needs for supportive care, the impact on employment opportunities and the problems experienced by older cancer survivors.

Various terms are applied to documents summarising cancer management, such as end of treatment summaries or individual survivorship care plans; these are vital to inform service design and research strategies. Such documents should provide a summary of treatment, potential late effects, guidance on follow up, indications for and route of re-referral and general and cancer-specific health promotion as well as a holistic needs assessment (HNA) [19]. These summaries may be perceived as time-consuming [20] and strategies are needed to automate these where possible, as a recent Macmillan survey indicated only 25% of respondents had received a summary document.

Different models of care will be required for cancer survivors, these may be initiated by secondary care in partnership with primary care and other organisations, but in most cases are likely to be based away from hospital care. Risk stratified pathways of care have been developed and piloted as part of the National Cancer Survivorship Initiative. Three basic levels have been suggested ranging from self management through shared care to complex specialist MDT managed care [20a]. ‘Self-management’ is a well-recognised strategy for the management of cancer as a long-term condition, by empowering cancer survivors to increase their self-efficacy, collaborating with the health care systems and other organisations to access appropriate and high-quality supportive care [21]. eHealth is likely to play a strong role in the development of cancer survivorship services; with positive effects reported for a variety of outcomes [22].

Poor lifestyle choices, such as inactivity, negatively impact on a range of global health measures. However, even in common cancers the evidence that modification of these choices has a significant impact is lacking, for example, there is some evidence of the impact of physical activity on recurrence rates in colorectal cancer [23] but these areas require much further investigation.

Concepts in survivorship, which are proving influential, include ‘survivorship navigation’ [24], the use of innovative electronic patient self-assessment [25] and the use of group visits [26]. However, interventions often lack robust scientific evaluation and are funded by third sector organisations, demonstrating the need for partnership to ensure scientific scrutiny to provide the most cost effective models of service delivery. Indeed, examples of third care partnerships are already well developed at a national level such as Macmillan and Department of Health (the National Cancer Survivorship Initiative, NCSI), and Scottish Government’s Transforming Care After Treatment (TCAT) programmes. These are developing and evaluating novel practice areas which include a well formulated recovery package, incorporating a holistic needs assessment, treatment summary at the end of each acute treatment phase shared with patient and GP, cancer care review performed by General Practice, and a patient education and support event (http://www.ncsi.org.uk/what-we-are-doing/the-recovery-package).

Cancer survivors require long term support in their communities. Hospital based services and oncologists may contribute usefully but integration with community and social care is essential. However, there appears to be barriers to widespread implementation, which need to overcome to facilitate roll out from these beacon sites. A priority must be to overcome barriers preventing widespread implementation of this type of comprehensive and joined up approach to management across all components of the health economy. Many pilot projects across the United Kingdom aim to encourage behaviour change but much more work is needed to look at sustainability, longer term measures of effect, how to address multiple unmet needs or behavioural change and the participation of groups who frequently have a low engagement, such as males, elderly and ethnic minorities.

Once again, the fields of childhood and young adult cancer survivorship [26a, 26b] leads the way with evidence-based guidelines under development as part of the EU-funded PanCareSurFup project (http://www.pancaresurfup.eu/), describing surveillance for important late effects of treatment (many written in collaboration with the International Late Effects of Childhood Cancer Guideline Harmonisation Group), and also the attributes of models of care including transition. This is timely since there is great interest in developing effective transition of long-term care during adolescence and young adulthood, thereby ensuring a successful move from paediatric to age-appropriate adult-led care. PanCare (the Pan-European Network for Care of Survivors after Childhood and Adolescent Cancer, http://www.pancare.eu/en/) is leading a multidisciplinary approach to improve, study, educate about and advocate for, childhood cancer survivorship care, which as it stands is an unmet service need that medical oncology may have a role in working in partnership with colleagues in the paediatric sector. Such partnerships may well provide a further model for the long term comprehensive management of survivors of adults cancer [27, 27a].

Lessons can be learned from paediatric oncology, though clearly the numbers involved in scaling up services for survivors of adults cancers, specific age-related toxicities that may be distinct, and integrating these with ageing and comorbidity will be significant challenges. The governmental model of integration of health and social care, alongside third sector partnerships, will be a vital strategic route for this emerging area.

Research in the area of survivorship will require shifts in our approach to study-design and a recognition of the need for protracted support for cancer survivors beyond routinely measured survival end points and beyond conventional hospital follow up. Cancer registries and other routinely collected health information may be useful in providing linkage of long-term data with outcomes from clinical trials, regarding late effects. The EORTC has developed a broad strategy for survivorship research and development [28]. While there are well recognised, though physician defined, QOL measures it is likely that evaluation of interventions and service developments for cancer survivors will be assessed using patient reported outcome measures (PROMs), which will drive the quality of survivorship care. An example is the national colorectal PROM survey in England that evaluated 21,000 individuals 12–36 months following diagnosis (NHS England http://www.england.nhs.uk/wp-content/uploads/2015/03/colorectal-cancer-proms-report-140314.pdf) with national and service provider level information provided (http://www.england.nhs.uk/resources/cancer-resources) [29].

Perhaps one of the most developed fields of enquiry, within which medical oncologists and the ACP have a close involvement is the follow-up of testicular cancer patients where there is a 95% 10-year survival rate. This has resulted in primary surveillance after surgical resection in early stage disease, placing a great importance upon concordance with structured follow-up. Where systemic therapy and radiation are required, there are large numbers of working-age long-term cancer survivors who are prone to second malignant neoplasms, toxicities to the kidneys and peripheral nerves and lungs, cardiovascular disease, reduced fertility and psychological and emotional problems, which are the subject of intensive research [30]. The issues in this patient group are relatively well defined and less complicated by co-morbidity, which means programmes or research in site-specific cancer may be easier to develop. However, due to the generic nature of many issues, a non-site-specific approach may also have a role. Increasing pressure on all health professionals could be a rate-limiting factor in the development of survivorship programmes. In fact, GPs may have more experience in managing multiple comorbidities and referral on to other agencies than secondary care staff. Indeed, we may be able to learn from pre-existing rehabilitation services, such as cardiac or respiratory.

The ACP will collaborate with other cancer organisations in a multidisciplinary dialogue with commissioners, patients’ representatives and primary care on new models of care for cancer survivors. Some medical oncologists will take a specialised interest in the management of cancer survivors. They will deliver some aspects of care and work with colleagues to develop services for cancer survivors. We anticipate components of patient education, self-management, GP involvement, specialist nursing, psychological support, pathway coordinators, providing specialist post-cancer care where evidence indicates it is beneficial and cost-effective. The approach will balance with community, multidisciplinary and appropriate specialist review in person, using patient-reported outcomes through telehealth and electronic means over time depending on needs. New models of care will have to incorporate specialist non-oncological expertise, such as endocrinology, nephrology, cardiology and psychology. Risk stratification, as increasingly practised in the long-term follow-up of childhood cancer survivors, and a similar graduated approach are required for the interventions to match the range of needs due to the limited staff and other resources.

In the future translational research may identify risk groups on a molecular basis and individual susceptibility, to allow targeted interventions [31].

The programme will undoubtedly change the nature of work in medical oncology over time, in keeping with our philosophy of response to changing patients’ needs.

### Actions

- Develop new oncology roles with special interest to develop services for cancer survivors
- Contribute to the Medical Oncology-led RCP conference on survivorship and late effects in 2016

## Figures and Tables

**Figure 1.** Cancer care trajectory and survivorship care. (Figure adapted from the Institute of Medicine. Reprinted with permission from *Cancer Patient to Cancer Survivor: Lost in Transition*, 2005, by the National Academy of Sciences, Courtesy of the National Academies Press, Washington, DC, USA and shown in Ganz [2].)

## References

[1] http://www.cancer.gov/dictionary?cdrid=450125.

[2] Ganz PA (2011). The ‘three Ps’ of cancer survivorship care. BMC Med.

[3] http://www.cancerresearchuk.org/cancer-info/cancerstats/survival/england-and-wales-cancer-survival-statistics.

[4] Oeffinger KC, Mertens AC, Sklar CA (2006). Chronic health conditions in adult survivors of childhood cancer. N Engl J Med.

[5] www.canceradvocacy.org.

[6] Skinner R, Wallace WH, Levitt GA (2006). Long term follow up of people who have survived cancer during childhood. Lancet Oncol.

[7] Armes J, Crowe M, Colbourne L (2009). Patients’ supportive care needs beyond the end of cancer treatment: a prospective, longitudinal survey. J Clin Oncol.

[8] Mehnert A (2011). Employment and work-related issues in cancer survivors. Crit Rev Oncol Hematol.

[9] Andrykowski MA, Manne SL (2006). Are psychological interventions effective and accepted by cancer patients? I. Standards and levels of evidence. Ann Behav Med.

[10] Lepore SJ, Coyne JC (2006). Psychological interventions for distress in cancer patients: a review of reviews. Ann Behav Med.

[11] http://www.wcrf.org/sites/default/files/Breast-Cancer-Survivors-2014-Report.pdf.

[12] Hudson MM, Ness KK, Nolan VG (2011). Prospective medical assessment of adults surviving childhood cancer: study design, cohort characteristics, and feasibility of the St. Jude lifetime cohort study. Pediatr Blood Cancer.

[13] Mols F, Thong MS, Vreugdenhil G (2009). Long-term cancer survivors experience work changes after diagnosis: results of a population-based study. Psychooncol.

[14] Pollack LA, Adamache W, Ryerson AB (2009). Care of long-term cancer survivors: physicians seen by Medicare enrollees surviving longer than 5 years. Cancer.

[15] Kero AE (2014). Cardiovascular morbidity in long-term survivors of early-onset cancer: A population-based study. Int J Cancer.

[16] Harrington CB, Hansen JA, Moskowitz M (2010). It’s not over when it’s over: long-term symptoms in cancer survivors - a systematic review. Int J Psychiatry Med.

[17] Bellury LM, Ellington L, Beck SL (2011). Elderly cancer survivorship: an integrative review and conceptual framework. Eur J Oncol Nurs.

[18] Brearley SG, Stamataki Z, Addington-Hall J (2011). The physical and practical problems experienced by cancer survivors: a rapid review and synthesis of the literature. Eur J Oncol Nurs.

[19] Mayer DK, Nekhyudov L, Snyder CF (2014). American society of clinical oncology expert statement on cancer survivorship care planning. J Oncol Pract.

[20] Dulko D, Pace CM, Dittus KL (2013). Barriers and facilitators to implementing cancer survivorship care plans. Oncol Nurs Forum.

[20a] Jefford M, Rowland J, Grunfeld E (2013). Implementing improved post-treatment care for cancer survivors in England, with reflections from Australia, Canada and the USA. Br J Cancer.

[21] McCorkle R, Ercolano E, Lazenby M (2011). Self-management: enabling and empowering patients living with cancer as a chronic illness Cancer J Clin.

[22] Ventura F, Ohlén J, Koinberg I (2013). An integrative review of supportive e-health programs in cancer care. Eur J Oncol Nurs.

[23] Otto SJ (2014). Association of change in physical activity and body weight with quality of life and mortality in colorectal cancer: a systematic review and meta-analysis. Support Care Cancer.

[24] Pratt-Chapman M, Simon MA, Patterson A (2011). Survivorship navigation outcome measures: a report from the ACS patient navigation working group on survivorship navigation. Cancer.

[25] Vickers AJ, Salz T, Basch E (2010). Electronic patient self-assessment and management (SAM): a novel framework for cancer survivorship. BMC Med Inform Decis Mak.

[26] Trotter K, Frazier A, Hendricks CK (2011). Innovation in survivor care: group visits. Clin J Oncol Nurs.

[26a] Robison LL, Mertens AC, Boice JD (2002). Study design and cohort characteristics of the Childhood Cancer Survivor Study: a multi-institutional collaborative project. Med Pediatr Oncol.

[26b] Hawkins MM, Lancashire ER, Winter DL (2008). The British childhood cancer survivor study: objectives, methods, population structure, response rates and initial descriptive information. Pediatr Blood Cancer.

[27] Woodward E, Jessop M, Glaser A (2011). Late effects in survivors of teenage and young adult cancer: does age matter?. Ann Oncol.

[27a] Glaser AW, Levitt G, Morris F (2013). Enhanced quality and productivity of long-term aftercare of cancer in young people. Arch Dis Child.

[28] Moser E, Meunier F (2014). Cancer survivorship: a positive side-effect of more successful cancer treatment. EJC Supplement.

[29] Downing A, Morris E, Richards M (2015). Health-related quality of life following colorectal cancer in England: a patient-reported outcomes study of 21,000 survivors 12–36 months post-diagnosis. J Clin Oncol.

[30] Travis LB, Beard C, Allan JM (2010). Testicular cancer survivorship: research strategies and recommendations. J Natl Cancer Inst.

[31] Visscher H, Ross CJ, Rassekh SR (2011). Pharmacogenomic prediction of anthracycline-induced cardiotoxicity in children J Clin Oncol.

# Brief report

**Published:** 2016

## 12. Precision oncology and genetics

**Dr Ellen Copson, Dr Zoe Kemp, Professor Diana Eccles, Professor Peter Johnson, and Dr Emily Shaw**

We will develop Precision oncology which will allow the development of cancer treatments with a greater probability of success and a lower probability of toxicity.

In 2001, the Secretary of State for Health noted that the NHS needed to ‘change and adapt its services’ to meet the challenge of genomics. Over the intervening years, research in both tumour and germline genetics has revolutionised our understanding of cancer biology and provided us with novel anticancer drugs. Thanks to the rapid development of sequencing technology, detailed genomic analysis is not only possible but is an increasingly cost effective part of modern oncological management. The increasing role of genomic medicine in routine care and the need for medical specialities to adapt training and working practices to ensure that patients receive optimal benefit from these advances has been highlighted in the PHG report ‘Genetics and mainstream medicine: Service development and integration’ [1].

### Tumour genomics

The completion of the Human Genome Project in 2000 heralded a new era in cancer biology. We now know that there are typically between 1000 and 10,000 somatic genetic changes in the genomes of most adult cancers. The identification of the key driver mutations in some tumour types has permitted the development of therapeutic agents which specifically target the aberrant protein product [2]. A number of these ‘targeted agents’ have fulfilled the promise of increased effectiveness and reduced toxicity in comparison to traditional cytotoxics and are now in routine clinical use whilst the rapid fall in cost of DNA sequencing technologies has made routine testing of tumour samples for specific mutations a viable option [2].

The feasibility of large-scale tumour genomic testing within the NHS has been demonstrated by the success of the first phase of the Cancer Research UK Stratified Medicine Programme in which >40,000 genetic tests were performed on over 9000 patient tumour samples at three central laboratories during a 2-year pilot study. Patient support for the concept of personalised cancer medicine was also confirmed: 10,750 patients consented to participate with a consent rate consistently in excess of 95% of those approached to participate [3].

Key issues that now need to be addressed by the oncology and pathology communities include the following:

- A need for robust data collection linking clinical phenotypes to tumour and germline genotypes and treatment response data, embedded into routine clinical care to facilitate clear genotype–phenotype correlation and help with interpretation of the clinical significance of genetic results [3]
- Establishment of agreed standards in molecular pathology including sample handling methods, nomenclature and reporting formats, turnaround times and integration of molecular and histopathological results [4, 5]
- Ensuring that relevant MDT professionals become conversant in the language of genomics, in order to understand what the results mean and their implications for management, as well as the relative merits and limitations of different techniques used for genetic analysis in different contexts (tumour versus blood, DNA versus RNA).
- The need to ensure that the cost of molecular testing and consequent clinical recommendations are rigorously assessed and appropriately funded by the NHS Commissioning Board or its equivalent in each nation of the United Kingdom.

The ACP will

- Support the work of organisations such as the Royal College of Pathologists, the Cancer Research UK Experimental Medicine Centres molecular pathology working groups, the Association of Clinical Pathologists molecular pathology committee and National External Quality Assurance Scheme for molecular pathology in establishing sample handling standards, performance indicators and reporting nomenclature for solid tumour somatic genetic analysis
- Support the work of the UK Genetic Testing Network Evaluation Group for germline genetic testing
- Promote the concept of standardised research consent for acquiring prospective and enduring patient consent for research use of tissue in routine NHS practice, based on national ethical standards for consent and information
- Encourage the development of clinical trials across multiple tumour sites with patients stratified by tumour molecular pathology features rather than by organ of origin or histological subtype

### Germline genetics

It is currently estimated that, approximately 3% of cancers arise on a background of germline mutations in cancer predisposition genes (CPGs) [6]. NHS testing for CPGs has been introduced gradually from the mid-1990s and has been delivered through a traditional clinical genetics model driven typically by a strong family history of cancer. Demand on the genetics service has increased steadily, with peaks of referral for testing sparked by greater public awareness from press reporting of high profile figures such as Angelina Jolie [7]. Increasing awareness that certain cancer phenotypes are associated with a significant risk of an underling CPG mutation, even in the absence of an obvious family history of malignant disease, have also increased the use of genetic testing. For example, the 2013 NICE guidelines on familial breast cancer 2013 recommend BRCA1/2 mutations testing for all young triple negative breast cancer patients regardless of family history [8].

Until recently, knowledge of an underlying CPG mutation mainly benefited relatives in better quantifying risk and opening options for targeted prevention. In addition, it has been used to inform breast cancer patients about future new primary cancer risk and facilitate decisions about risk reducing surgery; genetic testing has not otherwise altered the immediate management of the presenting malignancy. However, the advent of targeted therapies in the form of PARP inhibitors which exploit the underlying DNA repair deficiency in BRCA mutation carriers has now changed this paradigm [9]. Clinical trials of PARP inhibitors in adjuvant and metastatic settings have been successful and more are in progress. In addition, there is increasing evidence that mutations in CPGs may influence the effectiveness of certain chemotherapy drugs, with enhanced sensitivity to platinums reported in BRCA mutation carriers and reduced efficacy of 5FU in mismatch repair (MMR) gene mutation carriers [10]. Thus, there is now the potential for patient benefit from knowing their CPG mutation status at a much earlier stage than previously and advancements in technology including next generation sequencing have made ‘fast track testing’ a reality.

Traditionally, testing for CPG mutations has been performed only following referral to a clinical genetics service. However, increasing demands for testing in short time frames suggest that this model will not be sustainable in the future. An ‘oncogenetic’ model of CPG testing, whereby testing in patients with cancer can be performed through the cancer team, with support as required from genetics, is currently being piloted at several sites. The Royal Marsden hospital ‘Mainstreaming Cancer Genetics (MCG) programme’, (www.mcgprogramme.com) uses online teaching modules to train oncologists to inform and consent selected patients with ovarian and breast cancer for BRCA mutation testing [11]. Test results are communicated to the patient by a letter from the genetics service and if the test is positive, the patient is automatically sent an appointment by the genetics service to address future issues for the patient and implications for the family. The RMH has reported a very high patient satisfaction rate with this service [12].

However, the RMH MCG programme has incorporated a pre-specified format of laboratory reporting including clinical guidance and minimisation of reporting out of DNA variants. There is currently no standardised national reporting template or system of classifying variants that is linked to clear guidance on use of reported variants to inform clinical management [13]. A recent survey of breast cancer specialists confirmed a lack of knowledge in interpreting and communicating VUS reports [14].

It should also be noted that, unlike a somatic mutation, identification of a CPG germline mutation has implications for an entire network of relatives and not just for an individual. For a recently diagnosed cancer patient, struggling with cancer treatment and decisions around that, the added concern that they may have ‘passed’ on a pathogenic mutation to a child may be particularly distressing. It is therefore vital that appropriate support is available for patients who receive a positive mutation result. Patients with a negative BRCA result but very strong or complex family history will also still potentially benefit from a formal genetics review.

The ACP will:

- promote the development of mainstreaming genetic testing at appropriate oncology centres by providing training resources for both established consultant medical oncologists and doctors in postgraduate training and establishing models of care that promote multi-disciplinary working between oncologists and geneticists
- Contribute to the establishment of multidisciplinary training in genetics and cancer for scientists and clinicians working in oncology, genetics, and cellular pathology as part of postgraduate training programmes
- Support the work of the UK Genetic Testing Network Evaluation Group for germline genetic testing relevant to cancer
- work with clinical molecular genetics services to encourage the adoption by all genetics laboratories of a standard reporting template and a single classification system linked to clear clinical actions
- support the proposal by Genomics England for a central data collection repository, managed by the NHS, into which clinicians with patients’ consent could report unusual familial collections of cancers

### Training issues

Although the current medical oncology syllabus includes an appreciation of basic aspects of genomic medicine [15], exposure to clinical experience in cancer genetics is currently variable and frequently limited to out-of programme projects. The ACP has established an oncogenetic training working party in order to standardise and enhance the training of medical oncologists in oncogenetics and thus ensure that medical oncologists of the future are well placed to deal with the rapid advances in cancer genetics and mainstreaming genetics agenda. The OGWP has proposed four potential levels of oncogenetics training:

- A comprehensive understanding of basic genomics, including limitations of current technology, key differences between somatic and germline mutations and principles of stratified cancer medicine to be mandatory for all medical oncology trainees.
- Practical experience in cancer genetics clinics to be encouraged with the aim of providing medical oncologists who will be able to provide enhanced germline genetic advice in a MDT setting. It is envisaged that this would be available within many but not all regional training programmes and would be ideally linked to training in the most relevant tumour sites, for example, breast, ovarian, and colorectal cancer.
- More advanced experience in oncogenetics to be provided by formal post-CCT cancer genetics fellowships at a small number of tertiary centres for trainees who would like to develop a specialist interest in oncogenetics. It is envisaged that in the future there should be at least one medical oncology consultant at each tertiary centre with this level of experience in order to lead and coordinate this aspect of the medical oncology service.
- Dual accreditation in medical oncology and clinical genetics to be supported for appropriate trainees.

### Actions

- A workshop on precision oncology and genetic factors in oncology completed in October 2015 and will be followed by a text in preparation on Problem Solving through Precision Oncology in 2016/17.
- Development of the medical oncology training programme in genetics to include the following:
  - Review and enhance genomics content of medical oncology curriculum
  - Investigation of options for training to address any gaps in knowledge, skills, or competencies between those defined in the existing medical oncology curriculum and the minimum
  - Inclusion of cancer genetics experience as ‘optional’ clinical module in medical oncology training syllabus
  - Evaluation of options for linking in with existing or emerging Masters-level and other training courses in genetics being developed by NHS England in conjunction with the 100,000 genomes project
  - Evaluation of which clinical genetics centres may be willing to offer post CCT fellowships to medical oncology trainees and exploration of potential funding sources
- Evaluation and development of online support systems for precision practice for all oncologists to support the appropriate deployment of genetic testing and interpretation of results.

### Deliverables and monitoring

- Define the minimum required level of genetics knowledge required of all medical oncology trainees in conjunction with clinical genetics specialists.
- Review and expand medical oncology curriculum.
- Develop programme of online training resources in association with Genetics England and the MCG programme.
- Ascertain how many medical oncology registrars are currently able to undertake modules in genetics and which additional clinical genetics centres would be able to host oncology trainees via a survey of medical oncology and clinical genetics Training Programme Directors.
- Revise medical oncology clinical syllabus to include cancer genetics as ‘optional module’ and monitor uptake.

## Figures and Tables

**Figure 1.** Proposed new pathways to deliver cancer predisposition gene testing. (With thanks to Prof N. Rahman [11].)

## References

[1] Burton H (2011). Genetics and mainstream medicine PHG foundation.

[2] Stratton MR (2011). Exploring the genomes of cancer cells: progress and promise. Science.

[3] Johnson P CUK stratified medicine programme; solutions for nationwide delivery. http://www.cancerresearchuk.org/sites/default/files/smp1_booklet_1.2_-_no_marks.pdf.

[4] Gulley ML, Braziel RM, Halling KC (2007). Clinical laboratory reports in molecular pathology. Arch Pathol Lab Med.

[5] Cree IA, Deans Z, Ligtenberg MJ (2014). Guidance for laboratories performing molecular pathology for cancer patients European society of pathology task force on quality assurance in molecular pathology and the Royal college of pathologists. J Clin Pathol.

[6] Rahman N (2014). Realizing the promise of cancer predisposition genes. Nature.

[7] Evans DG, Barwell J, Eccles DM (2014). The FH02 study group, RGC teams, Miedzybrodzka Zosia, Murray Alex. The Angelina Jolie effect: how high celebrity profile can have a major impact on provision of cancer related services. Breast Can Res.

[8] (2013). Familial breast cancer: classification and care of people at risk of familial breast cancer and management of breast cancer and related risks in people with a family history of breast cancer.

[9] Fong PC, Boss DS, Yap TA (2009). Inhibition of poly(ADP-ribose) polymerase in tumors from BRCA mutation carriers. N Engl J Med.

[10] Turner NC, Tutt AN (2012). Platinum chemotherapy for BRCA1-related breast cancer: do we need more evidence?. Breast Cancer Res.

[11] Rahman N (2014). Mainstreaming genetic testing of cancer predisposition genes. Clin Med.

[12] http://mcgprogramme.com/news/accessed.

[13] Plon SE, Eccles DM, Easton D (2008). Sequence variant classification and reporting: recommendations for improving the interpretation of cancer susceptibility genetic test results. Hum Mutat.

[14] Eccles BK, Copson E, Maishman T (2014). Understanding of BRCA VUS genetic results by breast cancer specialists. BMC Cancer.

[15] Newton R, Farndon P (2013). Genomics in healthcare education—a review of national curricula. http://www.geneticseducation.nhs.uk/.
